# Population impact of lung cancer screening in the United States: Projections from a microsimulation model

**DOI:** 10.1371/journal.pmed.1002506

**Published:** 2018-02-07

**Authors:** Steven D. Criss, Deirdre F. Sheehan, Lauren Palazzo, Chung Yin Kong

**Affiliations:** 1 Institute for Technology Assessment, Massachusetts General Hospital, Boston, Massachusetts, United States of America; 2 Harvard Medical School, Boston, Massachusetts, United States of America; University of Pittsburgh, UNITED STATES

## Abstract

**Background:**

Previous simulation studies estimating the impacts of lung cancer screening have ignored the changes in smoking prevalence over time in the United States. Our primary rationale was to perform, to our knowledge, the first simulation study that estimates the health outcomes of lung cancer screening with explicit modeling of smoking trends for the whole US population.

**Methods/Findings:**

Utilizing a well-validated microsimulation model, we estimated the benefits and harms of an annual low-dose computed tomography screening scenario with a realistic screening adherence rate versus a no-screening scenario for the US population from 2016–2030. The Centers for Medicare and Medicaid Services (CMS) eligibility criteria were applied: age 55–77 years at time of screening, history of at least 30 pack-years of smoking, and current smoker or former smoker with fewer than 15 years since quitting. In the screened population, cumulative mortality reduction was projected to reach 16.98% (95% CI 16.90%–17.07%). Cumulative mortality reduction was estimated to be 3.52% (95% CI 3.50%–3.53%) for the overall study population, with annual mortality reduction peaking at 4.38% (95% CI 4.36%–4.41%) in 2021 and falling to 3.53% (95% CI 3.50%–3.56%) by 2030. Lung cancer screening would save a projected 148,484 life-years (95% CI 147,429–149,540) across the total population through 2030. There were estimated to be 9,054 (95% CI 9,011–9,098) overdiagnosed cases among the 252,429 (95% CI 251,208–253,649) screen-detected lung cancer diagnoses, yielding an overdiagnosis rate of 3.59%. The limitations of our study are that we do not explicitly model race or socioeconomic status and our model was calibrated to data from studies performed in academic centers, both of which may impact the generalizability of our results. We also exclusively model the effects of the CMS guidelines for lung cancer screening and not any other screening strategies.

**Conclusions:**

The mortality reduction and life-years gained estimated by this study are lower than those of single birth cohort studies. Single cohort studies neglect the changing dynamics of smoking behavior across generations, whereas this study reflects the trend of decreasing smoking prevalence since the 1960s. Maximum benefit could be derived from lung cancer screening through 2021; in later years, mortality reduction due to screening will decline. If a comprehensive screening program is not implemented in the near future, the opportunity to achieve these benefits will have passed.

## Abstract 2

Chung Yin Kong and colleagues model the health outcomes of lung cancer screening in the US and account for reduced levels of smoking. The benefits of screening and mortality reduction will start to decline in 5 years because of reduced smoking.

## Author summary

**Why was this study done?:**

- The National Lung Screening Trial demonstrated how low-dose computed tomography screening could reduce the burden of lung cancer mortality, currently the leading cause of cancer-related deaths in the US, and subsequent modeling studies have used trial data to extrapolate the long-term benefits and harms of lung cancer screening in the US.
- Those modeling studies were based on a single birth cohort (e.g., simulated individuals were all born in 1950) and thus ignored the effects of changing smoking behavior in more recent decades; thus, a more realistic estimation of the effects of a national lung cancer screening program was needed to better inform public health policy considerations.
- We performed, to our knowledge, the first lung cancer screening modeling study that takes into account decreasing smoking prevalence in the US, more accurately representing the effects of a screening program on the entire US population.

**What did the researchers do and find?:**

- We used mathematical modeling to project the benefits and harms of a national lung cancer screening program using a realistic screening adherence rate versus no screening in the US over the years 2016–2030.
- We projected that lung cancer mortality could be reduced by 3.52% and 148,484 life-years could be saved if lung cancer screening were implemented on a national scale.
- The screening program’s estimated rate of overdiagnosis, the primary harm of lung cancer screening, was 3.59% over the period, indicating the percentage of screen-detected lung cancers that had minimal negative consequences for the patient.
- Because of lower smoking prevalence in more recent years, our model predicted that the overall benefit of lung cancer screening on a national scale would peak in 2021 and then steadily decrease.

**What do these findings mean?:**

- Our study provides realistic projections of the time-sensitive benefits and harms of a national lung cancer screening program by accounting for changes in smoking behavior in the US, making our results useful for public health policy decision making.
- We have shown that since the benefits of screening are projected to decrease over time starting around 2021, there is a window of opportunity in the coming years for implementing a nationwide lung cancer screening program that can capture significant benefit.
- Our findings should be considered in light of limitations that affect the generalizability of our model—our results represent modeling of the US population at large without special considerations for racial or socioeconomic subpopulations, and we only evaluate the Centers for Medicare and Medicaid Services (CMS) guidelines as the screening strategy.

## Introduction

Despite more than 50 years having passed since the publication of the first Surgeon General’s Report on Smoking and Health and the expansive tobacco control efforts that have followed, lung cancer is still the leading cause of cancer-related deaths in the US [1,2]. This suggests that additional lung cancer control policies are necessary to work alongside tobacco control and cessation efforts in order to effectively reduce lung cancer deaths [3]. For example, lung cancer screening with low-dose computed tomography (CT) has been shown to improve early detection of lung cancer and reduce mortality among screened individuals [4,5]. The National Lung Screening Trial (NLST) resulted in a 20.0% reduction in lung cancer mortality for individuals at high risk of developing lung cancer when screened with low-dose CT compared to those screened with chest radiography [6]. The US Preventive Services Task Force (USPSTF) subsequently released a recommendation supporting low-dose CT screening [7], requiring private insurers to cover the cost of screening [8]. In 2015, the US Centers for Medicare and Medicaid Services (CMS) also issued a coverage determination for annual lung cancer screening with low-dose CT [9].

Several studies have utilized simulation modeling to estimate the effects of screening strategies on lung cancer mortality by following a single birth cohort over a number of years [10–13]. However, epidemiologic studies have shown that smoking behavior, as well as lung cancer risk, vary dramatically by birth cohort [14–17]. Thus, results of single birth cohort studies are not generalizable to the US at a population level. It is not known whether the screening benefit found by single-cohort simulations will remain after taking these birth cohort differences into account. The primary purpose of this study was to investigate this issue using a well-validated simulation model, the Lung Cancer Policy Model (LCPM), to estimate the impact of changing smoking prevalence on the number of lung cancer deaths, number of screening CT exams, overall mortality reduction, life-years gained, and overdiagnoses in the US at a population level using the CMS screening eligibility criteria and a realistic screening adherence rate. Additionally, we provide an estimate of the most beneficial timing for the implementation of a comprehensive lung cancer screening program.

## Materials and methods

### Model overview

Simulation modeling and clinical trials offer complementary methods of investigating the relationship between a healthcare intervention and outcomes. The advantage of simulation modeling is its ability to integrate currently available short-term trial data to project long-term consequences and explore a variety of “what-if” scenarios. For example, simulation modeling can explore different screening frequencies and alternative screening technologies while targeting different age, gender, and race groups. Simulation modeling can also be used to investigate topical questions in health policy discussions, such as the effect of varying rates of adherence on screening effectiveness.

The LCPM is a well-validated, comprehensive Monte Carlo microsimulation model of non-small cell lung cancer (NSCLC) and small cell lung cancer (SCLC) development, progression, detection, treatment, and survival [18,19]. The LCPM was previously utilized in lung cancer screening to study varied screening eligibility criteria, which informed the recommendations for lung cancer screening by the USPSTF [7,11].

The LCPM simulates progression, detection, follow-up, treatment, and survival of individual patients using a state-transition microsimulation method [20,21]. In the LCPM, lung cancer patients can develop adenocarcinoma (including adenocarcinoma in situ), large cell carcinoma, squamous cell carcinoma, small cell carcinoma, or other lung cancer. Histologic type informs lung cancer risks, incidence rates, and disease progression rates. Natural history parameters have been established through calibration to patient-level data from the NLST, and model outputs were validated using data from the Prostate, Lung, Colorectal and Ovarian Cancer Screening Trial (PLCO) [22]. We used the US Census Bureau’s population projections to estimate future lung cancer incidence [23].

Individual-level characteristics specific to the US population, such as age of smoking initiation, cigarettes smoked per day, and age of smoking cessation, were modeled using the “smoking history generator,” provided by the National Cancer Institute’s Cancer Intervention and Surveillance Modeling Network (CISNET) (S1 Appendix) [16,17,24]. All simulated individuals (current, former, and never smokers) aged 30–84 years were followed from 2016 to 2030. In order to estimate the variability from the stochastic nature of Monte Carlo microsimulation, we completed 20 runs of the simulation and reported means and 95% confidence intervals in our results.

Key inputs for the model include characteristics of the simulated population (e.g., birth year, smoking history, and smoking-adjusted mortality risk from competing causes) and scenario-specific items (e.g., test characteristics, screening program characteristics, screen adherence and eligibility, and response rates for treatments). For additional details, the model is described in an online technical appendix under the “MGH Institute for Technology Assessment” link on the CISNET Lung Cancer Model Profiles webpage (https://www.cisnet.cancer.gov/lung/profiles.html) and in S1 Appendix.

### Screening scenarios

In our evaluation of lung cancer screening using the LCPM, the control scenario was no lung cancer screening, while the comparison scenario was lung cancer screening criteria based on the CMS guidelines. Under the CMS screening criteria, current and former smokers aged 55–77 years with at least 30 pack-years of smoking history and fewer than 15 years since quitting were screened annually for lung cancer with CT. In these scenarios, CT screening was fully implemented in the year 2016 and studied up to the year 2030. Although the CMS screening guidelines were issued in early 2015 [9], we excluded the year 2015 in evaluating the screening impacts in order to capture the first full year of implementation.

Adherence is defined as the percentage of screening-eligible smokers who comply with the CMS screening guidelines. Adherent smokers are assumed to receive screening every year they are eligible, and nonadherent smokers are assumed to not receive screening. Studies of multiple programs have reported adherence rates to lung cancer screening ranging between 35% and 50%, though conclusive data on nationwide adherence were not available [25–27]. Additionally, adherence rates for more established screening programs for colorectal, breast, cervical, and prostate cancer were estimated to be 54.6%, 69.3%, 85.8%, and 46.4%, respectively, in 2010 by a national survey [28]. Given the nascent nature of the CMS lung cancer screening guidelines, it is unlikely that the adherence rate will exceed the adherence rates of these other screening programs. Therefore, in our base case analysis, we initially assumed 45% screening adherence to reasonably estimate potential benefits and harms of screening. This value represents a conservative estimate based on adherence data from more established screening programs in other cancers while also remaining within the range of observed rates in lung cancer screening studies.

Sensitivity analyses using 100% adherence and other lower adherence rates (i.e., 25% to 75%) considered the effect of screening adherence on lung cancer mortality reduction and deaths avoided. By estimating these key benefits at various screening adherence rates, we provide data that are necessary for health policy considerations and results that can serve as a tool to evaluate the impacts of existing screening programs. Our results assuming 100% adherence to lung cancer screening are presented in S3 Appendix and represent the maximum potential benefits and harms of a national lung cancer screening program.

Information on accessing US Census Bureau, NLST, and PLCO data, as well as additional information regarding the “smoking history generator,” used in this study can be found in S1 Text. We have also provided output data from the LCPM used in producing our results for this study in S1 Data.

## Results

### Outcomes in the absence of screening

Lung cancer cases were estimated to decrease 21.90% in the absence of screening to 141,114 (95% CI 140,934–141,294) annually in 2030 from 180,673 (95% CI 180,424–180,921) in 2016, resulting in cumulative lung cancer cases of 2,462,479 (95% CI 2,459,721–2,465,237) over the period. Former smokers made up an estimated 54.28% of these lung cancers, while current smokers made up 35.68%, and never smokers made up only 10.04%.

In the absence of screening, our model projected 1,777,144 (95% CI 1,775,138–1,779,151) cumulative lung cancer deaths between 2016 and 2030 for all birth cohorts in the US, with former, current, and never smokers totaling 952,578 (53.60% of total, 95% CI 950,983–954,173), 686,780 (38.65% of total, 95% CI 684,998–688,562), and 137,786 (7.75% of total, 95% CI 137,287–138,286) lung cancer deaths, respectively.

### Outcomes with screening

#### Total screens

**Fig 1:**
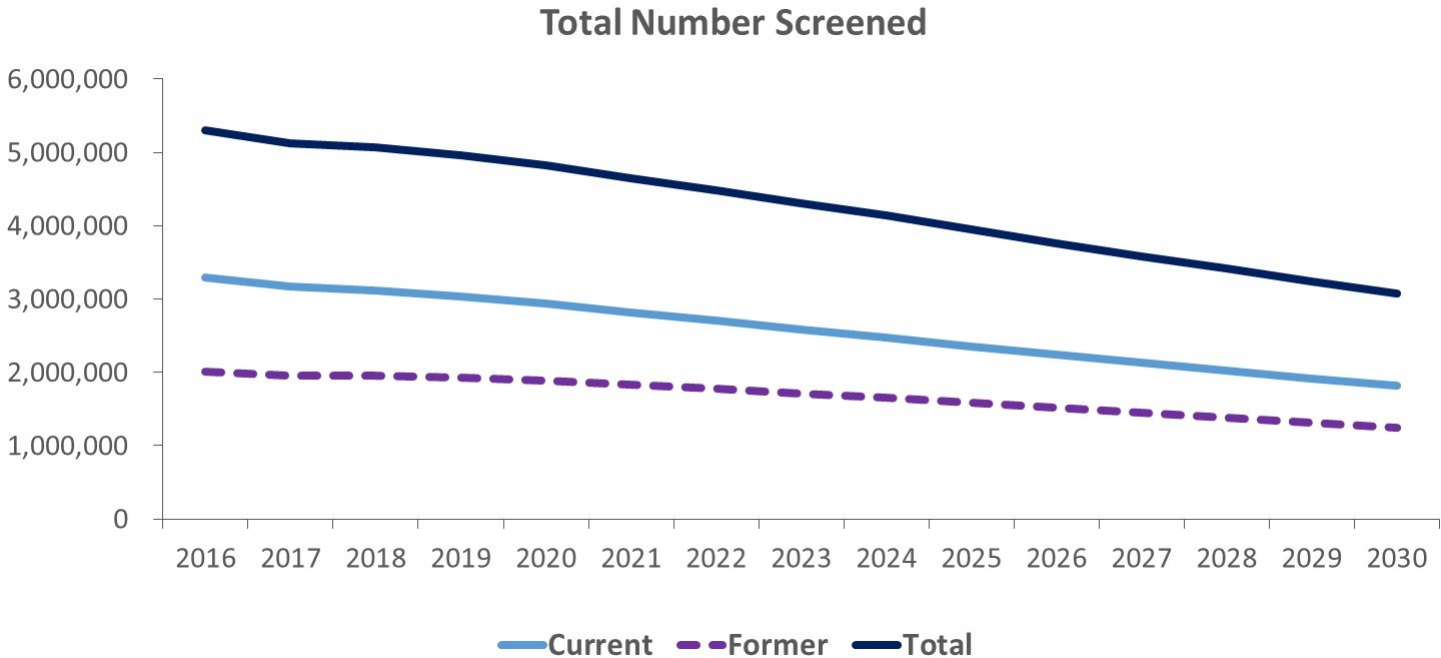
Projected total number of current and former smokers screened, 2016–2030.

Under the CMS screening eligibility criteria, cumulative screens through 2030 were projected to be 63,857,158 (95% CI 63,815,984–63,898,332). Fig 1 shows the total number of current and former smokers screened annually (Fig 1). Annual screens were estimated to decrease to 3,076,347 (95% CI 3,073,877–3,078,817) screens in 2030 (1.43% of study population) from 5,297,959 (95% CI 5,295,212–5,300,706) screens in 2016 (2.80% of study population). Throughout the study period, the majority of these simulated screens were of current smokers (decreasing slightly to 59.28% in 2030 from 62.12% in 2016); former smokers made up the remainder of these screens (increasing to 40.72% in 2030 from 37.88% in 2016).

Screening with an assumed 45% adherence rate in 2016, our estimates showed that current smokers screened made up 4.36% of the overall simulated population between the screening-eligible ages of 55 and 77 years, while former smokers screened made up only 2.66% of the same population. By 2030, these estimated percentages had decreased considerably to 2.10% and 1.44% for current and former smoker screens (S2 Appendix), respectively. Projected total screens decreased by 41.93% over the period.

#### Mortality reduction

**Fig 2:**
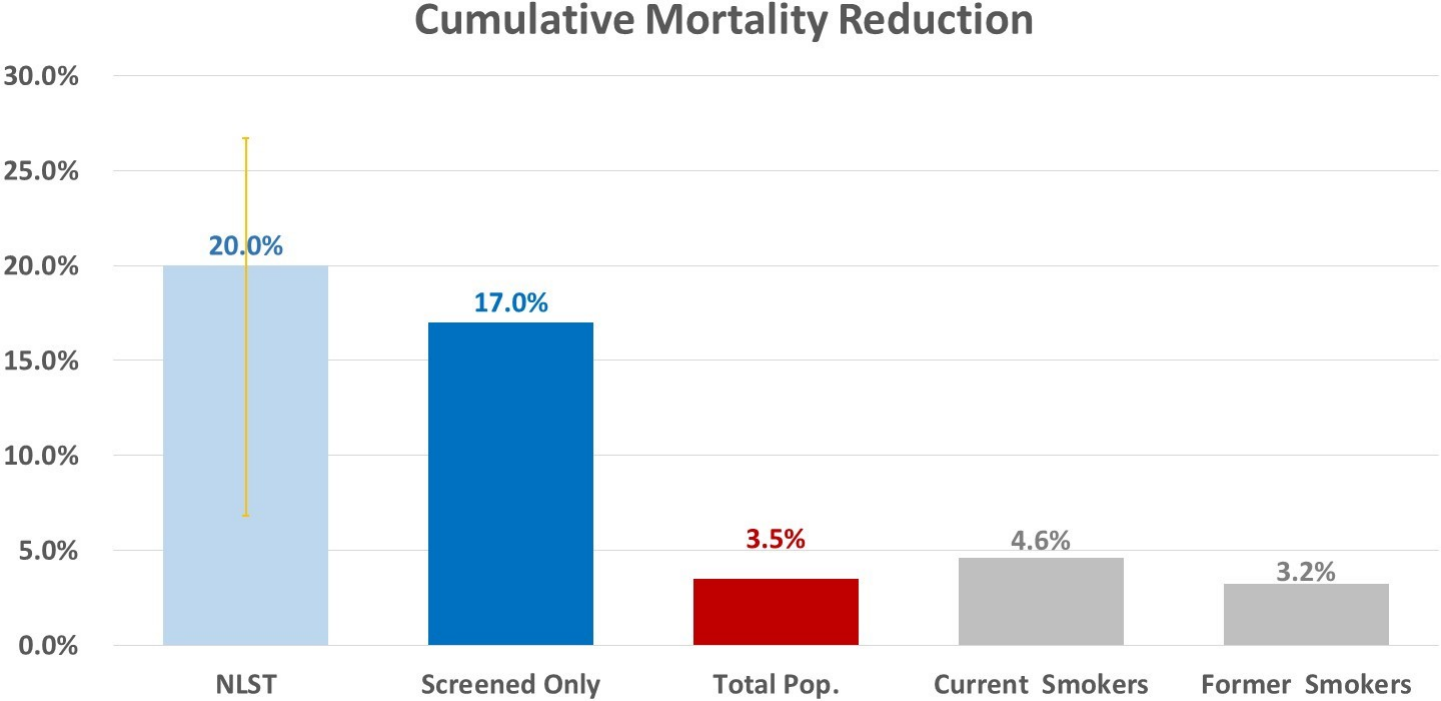
Projected cumulative mortality reduction comparison to National Lung Screening Trial (NLST) results, 2016–2030. Pop., population.

Mortality reduction is defined as the difference in lung cancer deaths between the no-screening and screening scenarios (i.e., deaths avoided), divided by the deaths in the no-screening scenario, giving the percentage of deaths in the no-screening scenario that could be avoided through screening. Cumulative lung cancer mortality reduction within the screened population (those individuals who were screened at least once) was estimated to be 16.98% (95% CI 16.90%–17.07%). Fig 2 compares lung cancer mortality reduction results from the NLST to those of the screened-only population, total population, and both smoker types in this study (Fig 2).

**Fig 3:**
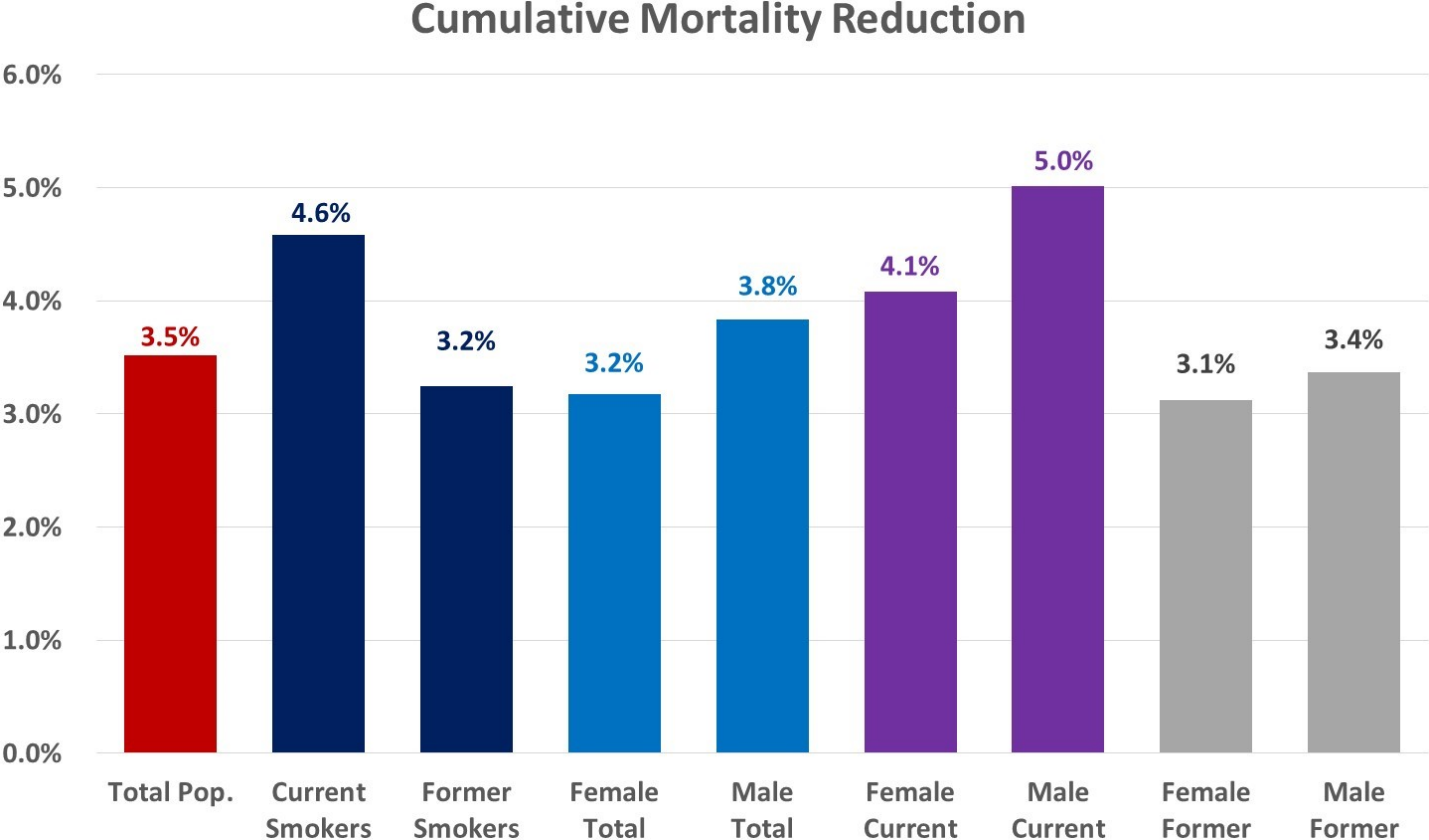
Projected cumulative mortality reduction stratified by smoker type and sex, 2016–2030. Pop., population.

The total cumulative mortality reduction across the study population, inclusive of those ineligible for screening under the CMS guidelines and nonadherent individuals, was estimated to be 3.52% (95% CI 3.50%–3.53%) by 2030. Cumulative mortality reductions for current smokers and former smokers were estimated to be 4.58% (95% CI 4.54%–4.62%) and 3.25% (95% CI 3.22%–3.27%), respectively. Fig 3 shows cumulative mortality reduction stratified by smoker type and sex (Fig 3).

**Fig 4:**
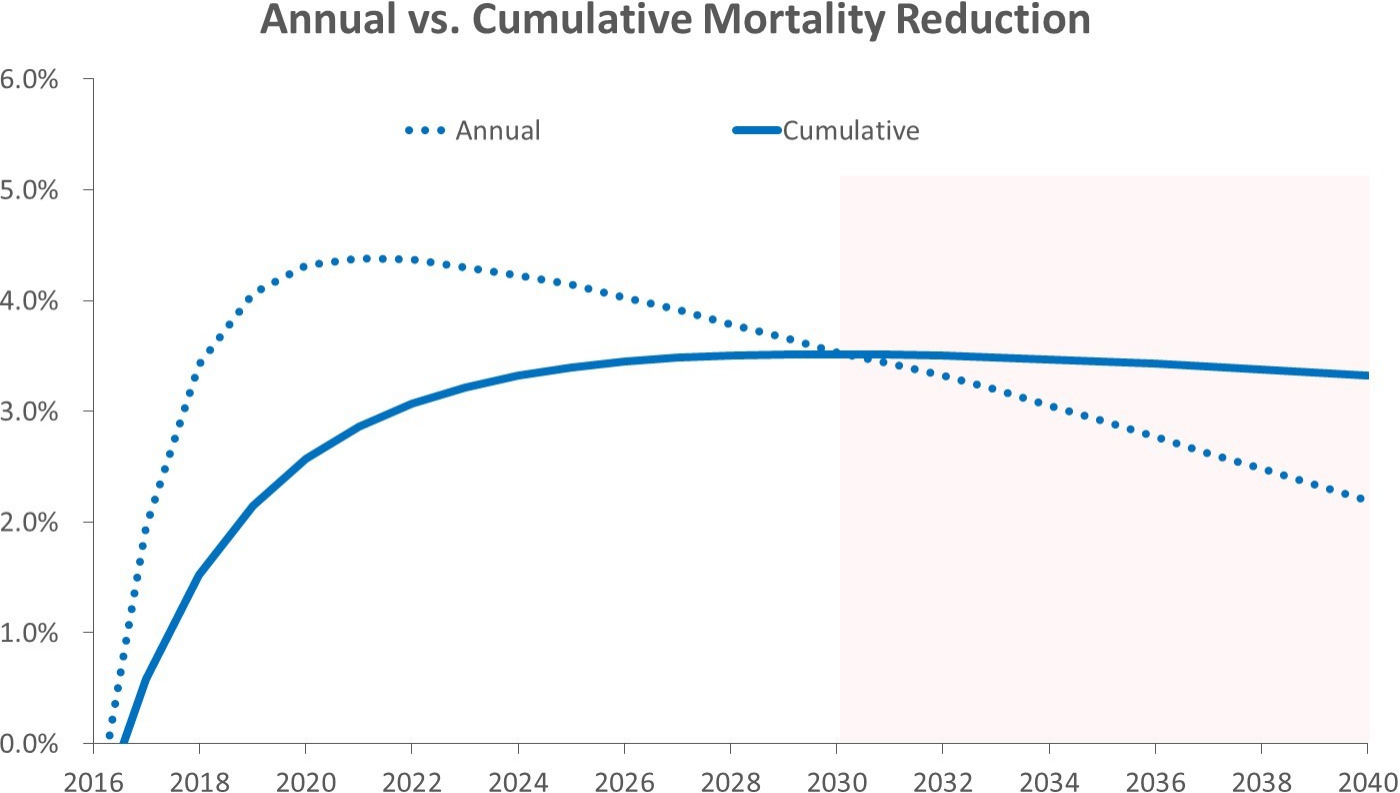
Projected mortality reduction for the total study population on an annual and a cumulative basis (extended past the study period to show trend).

Projected annual mortality reduction for the study population peaked in 2021 at 4.38% (95% CI 4.36%–4.41%) and decreased to 3.53% (95% CI 3.50%–3.56%) by 2030 (Fig 4). Current smoker mortality reduction on an annual basis reached an estimated maximum of 5.69% (95% CI 5.65%–5.74%) in 2021 before decreasing to 4.76% (95% CI 4.70%–4.81%) by 2030, and former smoker mortality reduction reached an estimated maximum of 4.03% (95% CI 4.00%–4.07%) in 2022 before decreasing to 3.37% (95% CI 3.33%–3.41%) by 2030 (S2 Appendix).

Estimated total deaths avoided amounted to 62,425 (95% CI 62,123–62,727), with current smokers accounting for 31,482 (95% CI 31,221–31,743) deaths avoided (50.43% of total) and former smokers accounting for 30,943 (95% CI 30,697–31,189) deaths avoided (49.57% of total) (Table 1). Our model predicted that the highest number of deaths could be avoided in the years 2021–2025, compared to the 5-year periods before and after.

**Table 1:** Estimated deaths avoided for current and former smokers compared to total population, 2016–2030.

Total deaths avoided by smoker type (95% CI)				
Smoker type	2016–2020	2021–2025	2026–2030	Cumulative
Current	8,861 (8,769–8,953)	12,892 (12,792–12,993)	9,728 (9,639–9,817)	31,482 (31,221–31,743)
Former	7,735 (7,674–7,796)	12,800 (12,689–12,910)	10,409 (10,310–10,508)	30,943 (30,697–31,189)
**Total**	**16,596** **(16,495–16,697)**	**25,692** **(25,571–25,814)**	**20,137** **(20,005–20,269)**	**62,425** **(62,123–62,727)**

We estimated that the highest proportion of total deaths avoided occurred between ages 70–74 years, 75–79 years, and 65–69 years (29.52%, 29.39%, and 21.33% of total, respectively; S2 Appendix). These age ranges also had the highest shares of projected total lung cancer deaths in the absence of screening, with individuals aged 75–79 years, 70–74 years, and 65–69 years composing 23.01%, 22.40%, and 16.90% of the total, respectively.

#### Mortality reduction for individual birth cohorts

**Fig 5:**
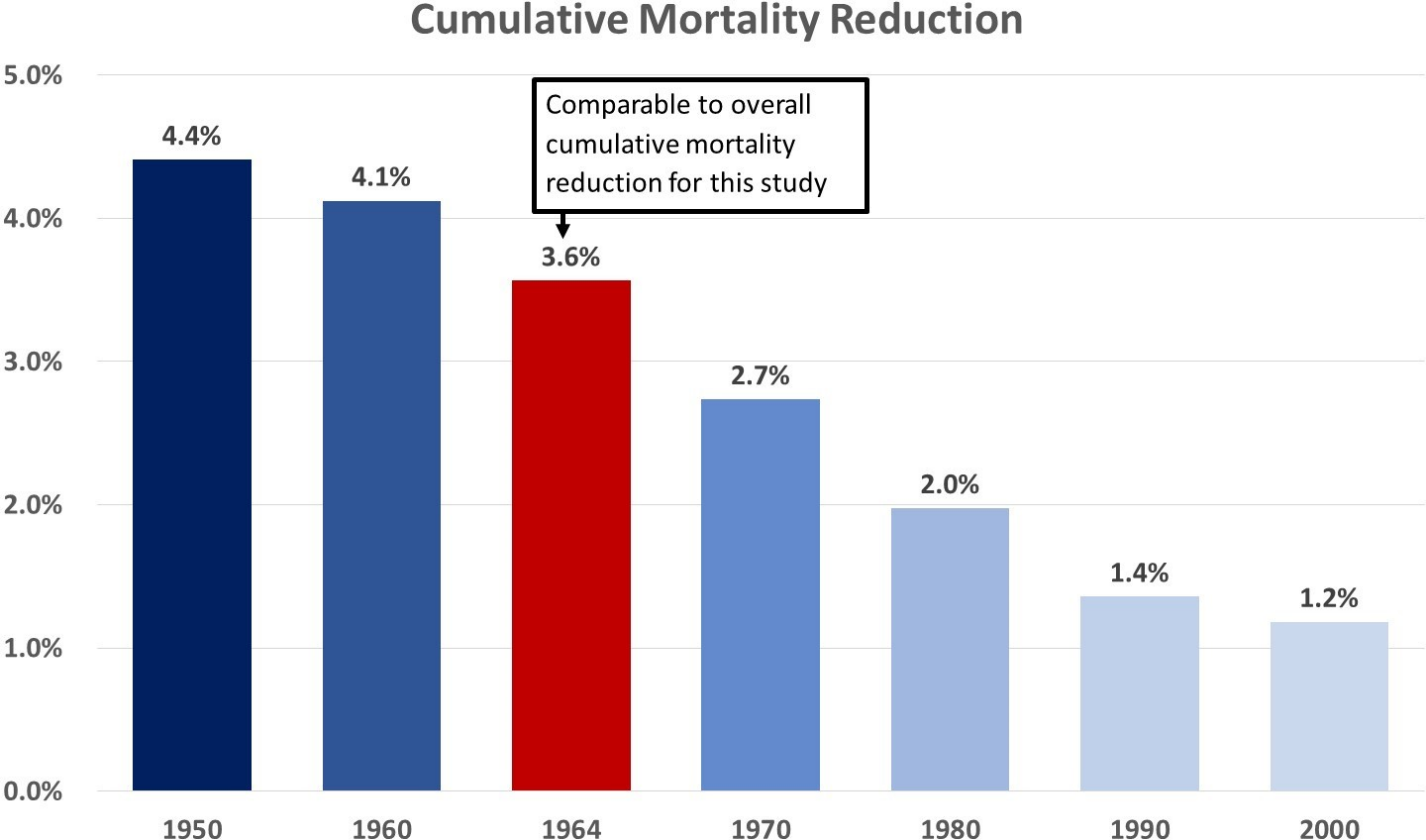
Projected cumulative mortality reduction for single birth cohorts.

We estimated the mortality reduction for individual birth cohorts to demonstrate how the benefits of screening can vary between birth cohorts. Mortality reduction was assessed over a 15-year period, with the observation period, in some cases, extending past the 2030 end year employed elsewhere in this study. The 1960, 1970, and 1980 birth cohorts had estimated cumulative mortality reductions of 4.12% (95% CI 4.10%–4.14%), 2.74% (95% CI 2.72%–2.75%), and 1.97% (95% CI 1.97%–1.98%), respectively. Projected mortality reduction trended downward for more recent birth cohorts, reflecting the overall trend of decreasing smoking prevalence in the US (Fig 5, S2 Appendix).

#### Life-years gained

Lung cancer screening with the CMS guidelines would save a projected 148,484 (95% CI 147,429–149,540) life-years across the total population through 2030. Simulated current smokers benefitted most in terms of life-years saved, with a total of 104,315 (95% CI 102,570–106,060), while simulated former smokers derived considerably less benefit in terms of life-years saved, with only 44,169 (95% CI 43,192–45,147) total.

#### Overdiagnoses

Overdiagnosed cases in this study are defined as those in which an individual is screened and subsequently diagnosed with lung cancer but eventually dies of causes other than lung cancer. These are deemed overdiagnoses because these additional lung cancer diagnoses that occur in the screening scenario compared to the no-screening scenario would have likely had limited negative consequences if they had remained undetected because of absence of screening [29].

Overdiagnosed cases were estimated to be 9,054 (95% CI 9,011–9,098), compared to an estimated 62,425 total deaths avoided via screening, yielding 14.50 overdiagnoses for every 100 deaths avoided. Compared to the 252,429 (95% CI 251,208–253,649) projected screen-detected lung cancer diagnoses, the estimated overdiagnosis rate was 3.59%. Patients aged 70–74 years made up the largest portion of these overdiagnoses with an estimated 3,417 (95% CI 3,400–3,433), while patients aged 75–79 years also made up a significant amount of the overdiagnoses with an estimated 3,196 (95% CI 3,181–3,212), together making up 73.04% of all projected overdiagnoses (S2 Appendix).

We also compared overdiagnoses in our study to those estimated in the NLST. Patz et al. previously developed an excess cancer diagnosis rate considering a public health perspective to estimate the true level of overdiagnosis in the NLST [29]. This definition takes the difference in the number of lung cancer cases between the low-dose CT screening arm and the chest X-ray (control) arm and divides this number by the total number of lung cancers diagnosed in the low-dose CT arm [29]. This calculation provides the percentage of lung cancer diagnoses in the low-dose CT arm that are in excess of those that would have been diagnosed through clinical presentation or other means without screening. Using this method with our no-screening scenario as the control and 100% adherence (given adherence in the NLST was nearly 100%), our overdiagnosis rate was estimated to be 9.46% (95% CI 9.42%–9.51%), comparable to the 11.0% result of the Patz study [29]. S2 Appendix provides a comparison of these 2 methods for different age ranges.

#### Impacts of screening adherence

Reported adherence rates vary between different studies and will likely have a considerable impact on the efficacy of screening. To quantify the potential impact of adherence, we estimated the cumulative lung cancer mortality reduction and deaths avoided for the total population with screening adherence rates of 25% to 75% and perfect adherence (100%), given the inconclusiveness of published data on the matter (Table 2). Mortality reduction in the perfect adherence scenario was estimated to be 7.81% (95% CI 7.77%–7.85%), and an estimated 138,722 (95% CI 138,052–139,393) deaths were avoided, representing a missed opportunity to avoid approximately 76,297 more lung cancer deaths if all eligible patients participated in screening, compared to the 45% adherence rate used in this study. If a national lung cancer screening program were able to attain an adherence rate of 75%, comparable to breast and cervical cancer screening (69.3% and 85.8%, respectively), our model predicted a mortality reduction of 5.86% (95% CI 5.83%–5.89%) and 104,042 (95% CI 103,539–104,545) deaths avoided.

**Table 2:** Sensitivity analysis for overall cumulative mortality reduction and total deaths avoided estimates by adherence rate.

Sensitivity analysis: Adherence rate (95% CI)		
Rate	Mortality reduction	Deaths avoided
25%	1.95% (1.94%–1.96%)	34,681 (34,513–34,848)
35%	2.73% (2.72%–2.75%)	48,553 (48,318–48,788)
**45%**	**3.52% (3.50%–3.53%)**	**62,425 (62,123–62,727)**
55%	4.30% (4.28%–4.32%)	76,297 (75,928–76,666)
65%	5.08% (5.05%–5.10%)	90,170 (89,734–90,606)
75%	5.86% (5.83%–5.89%)	104,042 (103,539–104,545)
**100%**	**7.81% (7.77%–7.85%)**	**138,722 (138,052–139,393)**

## Discussion

Using the LCPM, we performed, to our knowledge, the first multiple birth cohort simulation study to estimate the health outcomes of lung cancer screening for the whole US population. Over the period 2016–2030, our model estimated that the number of people eligible for screening would decrease from 11,773,243 (95% CI 11,767,138–11,779,347) to 6,836,326 (95% CI 6,830,837–6,841,815). During this period, cumulative lung cancer mortality reduction was estimated to be 3.52% and 16.98% for the total population and screened individuals, respectively. The estimated lung cancer mortality reduction within the screened population was similar to the observed result of 20.0% in the NLST. Our study predicts that lung cancer screening at a population level would yield 14.50 overdiagnosed cases for every 100 deaths avoided and 3.59 overdiagnosed cases for every 100 cases of screen-detected lung cancer. We used a screening adherence rate of 45% in our base case scenario in order to provide realistic estimates of the benefit of a fully implemented lung cancer screening program. Compared to the results using an ideal adherence rate of 100%, the more practical rate used in our study demonstrates how improving the adherence rate is pivotal to the success of a population-level screening program.

### Comparison to single birth cohort studies

Prior lung cancer screening modeling studies that followed a single birth cohort (e.g., a US cohort born in 1950) and used similar eligibility requirements and 100% adherence reported estimated mortality reduction to be approximately 14%—higher than our reported estimate of 7.81% for the US population with 100% adherence [10,11]. A potential mechanism for the difference in mortality reduction is the continuous turnover of eligible smokers in our multiple birth cohort analysis. Earlier birth cohorts with higher smoking prevalence (i.e., cohorts born in the 1950s and early 1960s) experience a higher lung cancer mortality reduction because there are more high-risk smokers eligible for screening and, thus, more opportunity for early detection of lung cancers. When single birth cohort analyses estimate lung cancer mortality reduction, they only capture the benefit at its maximum—with just 1 cohort that has considerably higher risk of lung cancer than do later cohorts. Therefore, the results of single birth cohort analyses are not generalizable to the US population as a whole.

In our study, simulated individuals from more recent birth cohorts with lower smoking prevalence are replacing these older smokers each year, resulting in a lower percentage of the study population eligible for screening. This can be seen by comparing the estimated 19% of the 1950 cohort eligible for screening in prior single cohort studies to the estimated 6.22% of our study population eligible in the first year of our analysis, when the most individuals from earlier cohorts are present [10,11]. The percentage eligible decreases to 3.17% over the study period, as more recent cohorts with lower smoking prevalence enter the study population.

Fewer eligible individuals translates into lower mortality reduction in later birth cohorts, as there are both fewer smokers being screened and fewer lung cancers to be detected. The results of our single birth cohort analyses provide a validation of this mechanism, showing higher estimated mortality reduction for earlier US cohorts (Fig 5, S2 Appendix). Our lower estimated mortality reduction is, therefore, a product of lower risk birth cohorts partially offsetting the higher benefit derived by earlier birth cohorts.

Studying multiple birth cohorts over an extended period of time captures changes in smoking prevalence and gives a practical view of the benefit of screening for public health policy decision making. Single cohort simulation studies can be more easily implemented, but they neglect smoking behavior trends over different generations by providing only the benefits and harms for 1 cohort. Smoking prevalence strongly influences lung cancer incidence cases and deaths [30,31], thereby highlighting the need for an evaluation of lung cancer screening that considers the proportion of current and former smokers in the total population over time, as in this study.

### Limitations

Because our study is meant to be an analysis of the US population at large, there is no explicit modeling of race or socioeconomic status in our projections of the health outcomes of lung cancer screening. Significant variation exists in the smoking behaviors of different races and individuals of different socioeconomic status, which can influence lung cancer incidence rates and access to care within these populations [32–35]. Due to these factors, individuals in these populations may not receive proportionate benefit, and therefore, our results may not be generalizable to particular stratums within the US population. While projecting the effects of screening for these subpopulations would be valuable, the LCPM does not currently have the capability to model US smoking behavior at this level of detail (i.e., with stratifications by race and socioeconomic status). However, the development of a more comprehensive model to estimate screening effects for these subpopulations is currently the focus of ongoing research by the CISNET consortium.

An additional limitation of this study is that our model was calibrated to data from the NLST and PLCO [22]. These studies were performed in academic centers and may have enrolled healthier individuals in better-organized screening programs compared to the average community health center. Therefore, the conditions under which these studies were executed may not be representative of screening programs implemented at health centers across the country. These differences between academic trials and screening programs at the average community health center could translate to slightly lower overall effectiveness when considering a national screening program. The primary driver of this lower effectiveness would likely be reduced screening adherence due to lower quality healthcare infrastructure and more patients with serious comorbidities being unable to receive screening. These differences are taken into account in the 45% adherence rate used for this study and are further explored in the sensitivity analysis on screening adherence rate, though other differences in the quality of facilities, providers, and treatments could also impact effectiveness.

Finally, our study is limited in that it is confined to the guidelines issued by the CMS. We do not evaluate whether a better screening strategy for lung cancer mortality reduction exists in comparison to the CMS guidelines but rather estimate the national impact of the current guidelines issued by the CMS. In doing so, the eligible age in our study extends to 77 years, which could include individuals who are technically eligible based on smoking history and age but would not be screened because of comorbidities.

### Implications

Although lower than the mortality reduction estimated by single cohort studies, our study’s projected mortality reduction demonstrates the potential efficacy of screening with low-dose CT for the many birth cohorts that constitute the US population if adherence rates are improved. Further demonstrating lung cancer screening’s value to the US population, screening was projected to save 148,484 life-years. The estimated harms—mainly overdiagnoses—associated with screening were minimal compared to these potential benefits. While overdiagnoses cause patients to endure unnecessary treatment, morbidity, cost, and potential anxiety, only in very rare cases do they cause death [29,36]. Therefore, the estimated benefits of lung cancer screening on a population level would likely outweigh these harms attributable to overdiagnosis.

With an estimated 138,722 deaths that could be avoided by 2030 with 100% adherence, lung cancer screening using the CMS guidelines provides an opportunity for substantial benefit to the US population yet remains challenged by the need for improving and maintaining adherence to screening. With the resources and guidelines available to implement a comprehensive screening program in the US, greater efforts must be made to reach high-risk smokers and make known the advantages and limited downsides of participating in screening. The projected impact of adherence on screening effectiveness shown in our study highlights this need for more comprehensive action to raise awareness and encourage adherence to guidelines [9,37,38].

While screening has been shown to reduce lung cancer mortality, decreasing smoking prevalence in more recent generations will eventually lower the effectiveness of a nationwide screening program. Our analysis of lung cancer screening at a population level illustrates the window of opportunity to secure significant benefit for those with lung cancer and when that window may close. Based on our projections, annual mortality reduction will peak in the next 5 years before declining because of lower smoking rates in younger birth cohorts. Therefore, the time frame for organizing and executing a strategy for nationwide lung cancer screening would ideally be in the coming years. Delays on fully implementing lung cancer screening will forfeit the opportunity to prevent more than 62,000 lung cancer deaths and minimize the burden of lung cancer in the next 15 years. As smoking prevalence declines, the effectiveness of a large-scale screening program, for the most part, becomes less favorable, and a change to the current policies regarding lung cancer control will be warranted.

While lung cancer screening is not seen by some as a health policy priority [39], the results demonstrated in this study should spur further conversation into how we can better use early detection to reduce the deadly consequences of lung cancer.

## Supporting information

S1 Appendix Additional detail on the Cancer Intervention and Surveillance Modeling Network’s smoking history generator and information regarding the Lung Cancer Policy Model. (PDF)

S2 Appendix Supplemental figures to accompany the primary results. (PDF)

S3 Appendix Results and figures assuming a 100% screening adherence rate. (PDF)

S1 Data Output data from the Lung Cancer Policy Model used in producing our results for this study. (CSV)

S1 Text Information on accessing US Census Bureau, National Lung Screening Trial (NLST), and Prostate, Lung, Colorectal and Ovarian Cancer Screening Trial (PLCO) data, as well as additional information regarding the smoking history generator, used in this study. (DOCX)

## Abbreviations

CISNET: Cancer Intervention and Surveillance Modeling Network
CMS: Centers for Medicare and Medicaid Services
CT: computed tomography
LCPM: Lung Cancer Policy Model
NLST: National Lung Screening Trial
NSCLC: non-small cell lung cancer
PLCO: Prostate, Lung, Colorectal and Ovarian Cancer Screening Trial
SCLC: small cell lung cancer
USPSTF: US Preventive Services Task Force
