# Supplementary material for: Population impact of lung cancer screening in the United States: Projections from a microsimulation model
Source: PLoS Med. 2018 Feb 7;15(2):e1002506. doi: 10.1371/journal.pmed.1002506 (PMC5802442; doi:10.1371/journal.pmed.1002506)
Supplement: S1 Appendix — (PDF) [file pmed.1002506.s001.pdf]

S1 Appendix

Population Impact of Lung Cancer Screening in the United States

Supplementary Materials

Contents

Page 3 – **Smoking History Generator:** Additional detail on the Cancer Intervention and Surveillance Modeling Network's smoking history generator

Page 4 – **Lung Cancer Policy Model:** Additional detail on the parameters and operations of the Lung Cancer Policy Model

Page 5 – **Lung Cancer Policy Model Schematic**

Smoking History Generator

Additional detail on the Cancer Intervention and Surveillance Modeling Network's smoking history generator

Background

The National Cancer Institute's Cancer Intervention and Surveillance Modeling Network developed the smoking history generator (SHG) in order to provide smoking history and other cause mortality inputs for lung cancer models. The smoking history generator provides cohort-specific smoking histories, as well as other cause death rates to be used in simulation modeling analyses of lung cancer interventions, including screening and tobacco control. The SHG has been used to estimate the impact of tobacco control on U.S. smoking-related mortality since the publication of the 1964 Surgeon General's Report [1] and to estimate the health effects of raising the minimum purchase and sale age of tobacco products in the U.S. [2].

Determination of Parameters

The methods for deriving the model parameters and extrapolating the SHG's output to calendar year 2030 have been previously described [3]. The data sources of the SHG include the National Health Interview Survey (NHIS), the Human Mortality Database (HMD) life tables, and the Cancer Prevention Study (CPS)-I and II [3-6].

The parameters in the SHG were estimated by fitting the data sources with an age, period, and cohort model. The smoking data from the NHIS was smoothed to provide information by gender, age, and year on the mean and variability in smoking status (never, current, and former by years quit), intensity, and duration. The HMD life tables were used to derive life tables for other-cause mortality associated with specific levels of smoking. The smoking related parameters, including initiation and cessation rates, were fitted using generalized linear models. The cigarettes smoked per day were fitted using a cumulative logistic model with constrained splines for temporal effects. To extrapolate the smoking prevalence and intensity to year 2030, the SHG used the latest available data of birth cohort in 1979 and kept all model parameters constant.

Lung Cancer Policy Model

Additional detail on the Lung Cancer Policy Model

Background

The LCPM is a Monte Carlo microsimulation model of lung cancer development, detection, and treatment coded in C++. Simulated patients progress through five potential model states: 1) General Population, 2) Follow-Up, 3) Diagnosis & Staging, 4) Treatment, and 5) Death.

Overview of Process

Initially, the LCPM is populated with healthy, disease-free individuals who enter into the aforementioned model states based on monthly transition probabilities. The likelihood of developing lung cancer is tied to an individual's smoking history, while non-smokers are also capable of developing lung cancer from other causes. During each simulated month, individuals may develop lung cancer, experience growth of an existing cancer, or develop metastases and other symptoms.

Once a patient has developed lung cancer, it can be detected through three different means: clinical presentation due to symptoms, incidental imaging, or low-dose CT screening. Screen-detected lung cancers will experience different behavior. Next, patients receive diagnostic testing and are evaluated for staging, possibly undergoing treatment based on the outcomes of those activities.

Simulating Lung Cancer Development and Progression

An individual can develop a maximum of three cancers and monthly probabilities of developing lung cancers are estimated using an independent logistic equation based on cancer cell type. For each histologic type, the logistic equation utilizes specific intercept, cell type-specific coefficients for age, age^2, years of cigarette exposure (smoke-years, SY), an interaction term between SY and age^2, average number of cigarettes smoked per day, and years since quitting smoking. Growth of malignant tumors is governed by a Gompertz function where doubling times decrease with an increase in tumor size. Cancer progression over time is modeled to include nodal involvement and/or distant metastases.

Model Calibration and Validation

Potential lung cancer cell types include non-invasive and invasive adenocarcinoma, large cell, squamous cell, small cell, and other. Natural history parameters were obtained from de-identified data from the National Lung Screening Trial (NLST) and Prostate, Lung, Colorectal and Ovarian (PLCO) screening trial participants, with outputs calibrated and validated to these studies [7]. A detailed description of the original LCPM is publicly available, as recorded within a designated National Cancer Institute website (http://www.cisnet.cancer.gov/lung/profiles.html).

Lung Cancer Policy Model

Lung Cancer Policy Model Schematic

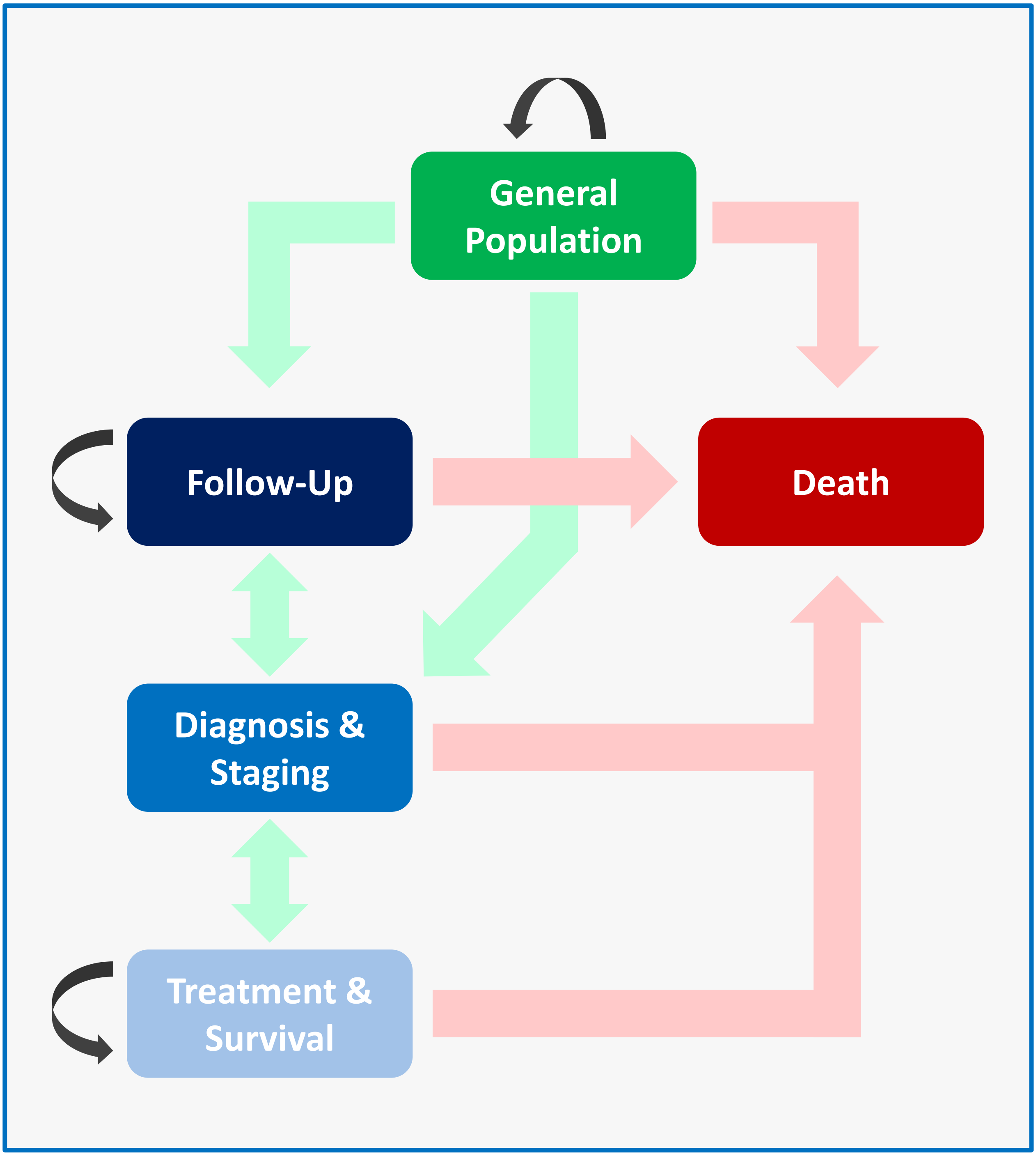

References

1. Moolgavkar SH, Holford TR, Levy DT, et al. Impact of reduced tobacco smoking on lung cancer mortality in the United States during 1975-2000. *J Natl Cancer Inst.* 2012;104(7):541-548.

2. Institute of Medicine. Health Implications of Raising the Minimum Age for Purchasing Tobacco Products. 2014. http://www.nationalacademies.org/hmd/Activities/PublicHealth/TobaccoMinimumAge.aspx. Accessed 9/6/2017

3. Jeon J, Meza R, Krapcho M, Clarke L, Byrne J, Levy DT. Actual and Counterfactual Smoking Prevalence Rates in the US population via Micro-simulation. *Risk analysis : an official publication of the Society for Risk Analysis*. 2012;32(Suppl 1):S51-S68. doi:10.1111/j.1539-6924.2011.01775.x.

4. Holford TR, Levy DT, McKay LA, Clarke L, Racine B, Meza R, et al. Patterns of birth cohort-specific smoking histories, 1965-2009. Am J Prev Med. 2014;46(2):e31-7.

5. Barbieri M, Wilmoth JR, Shkolnikov VM, Glei D, Jasilionis D, Jdanov D, et al. Data Resource Profile: The Human Mortality Database (HMD). Int J Epidemiol. 2015;44(5):1549-56.

6. Calle EE, Rodriguez C, Jacobs EJ, Almon ML, Chao A, McCullough ML, et al. The American Cancer Society Cancer Prevention Study II Nutrition Cohort: rationale, study design, and baseline characteristics. Cancer. 2002;94(2):500-11.

7. Meza R, ten Haaf K, Kong CY, Erdogan A, Black WC, Tammemagi MC, et al. Comparative analysis of 5 lung cancer natural history and screening models that reproduce outcomes of the NLST and PLCO trials. Cancer. 2014;120(11):1713-24.
