# Supplementary material for: Population impact of lung cancer screening in the United States: Projections from a microsimulation model
Source: PLoS Med. 2018 Feb 7;15(2):e1002506. doi: 10.1371/journal.pmed.1002506 (PMC5802442; doi:10.1371/journal.pmed.1002506)
Supplement: S2 Appendix — (PDF) [file pmed.1002506.s002.pdf]

S2 Appendix

Population Impact of Lung Cancer Screening in the United States

Supplementary Figures for Base Case

Contents

Page 3 – **Supplementary Figure 1:** Percent of population screened by smoker type among 55-77 year-olds

Page 4 – **Supplementary Figure 2:** Current and former smoker mortality reduction on annual and cumulative basis

Page 5 – **Supplementary Figure 3:** Share of cumulative deaths avoided stratified by age range, 2016-2030

Page 6 – **Supplementary Figure 4:** Cumulative mortality reduction for single birth cohorts

Page 7 – **Supplementary Figure 5:** Comparison of the Patz method of calculating overdiagnoses and the alternative method used in our study

Page 8 – **Supplementary Figure 6:** Study population stratified by smoker type

Supplementary Figure 1

Percent of population screened by smoker type among 55-77 year-olds.

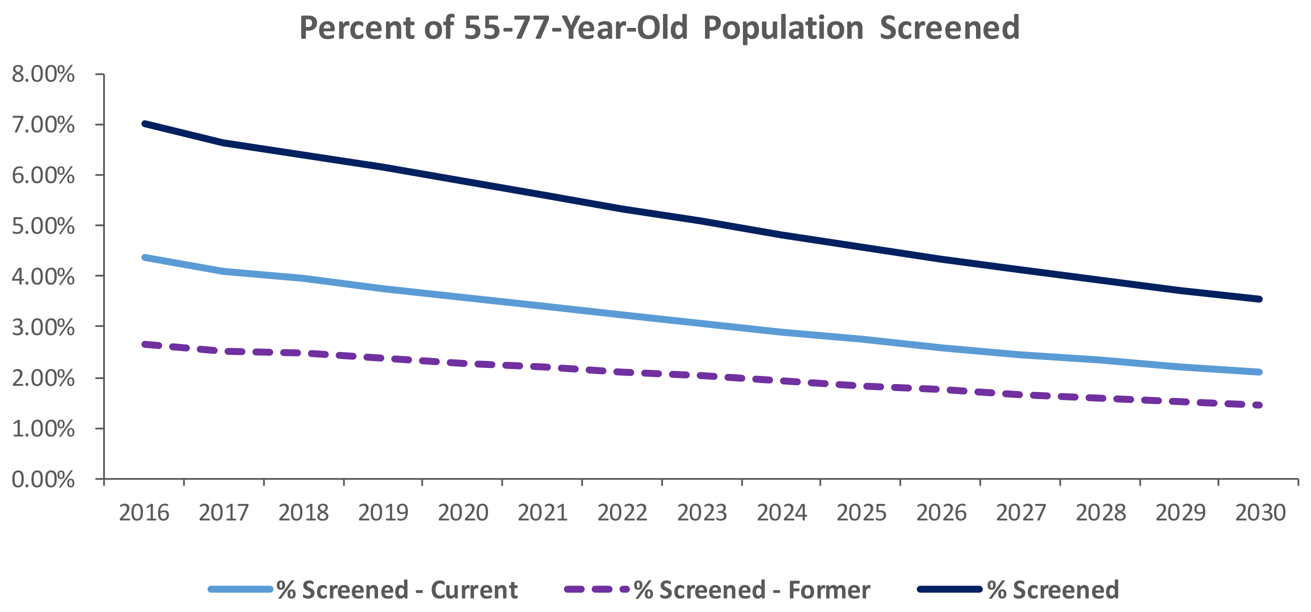

Supplementary Figure 1. The annual decline in the percentage of individuals within the screening-eligible age who receive screening suggests the significant effect of changing smoking behavior in the U.S. As later cohorts enter the study, their lower smoking prevalence causes fewer individuals to meet the eligibility requirements set out by the CMS and, thus, a higher proportion of the screening-eligible age group does not receive screening. This directly impacts mortality reduction, as having a lower proportion of individuals being screened reduces the opportunity for lung cancers to be detected. The lower mortality reduction can also be a function of lighter smoking habits leading to the development of fewer lung cancers.

Supplementary Figure 2

Current and former smoker mortality reduction on annual and cumulative basis.

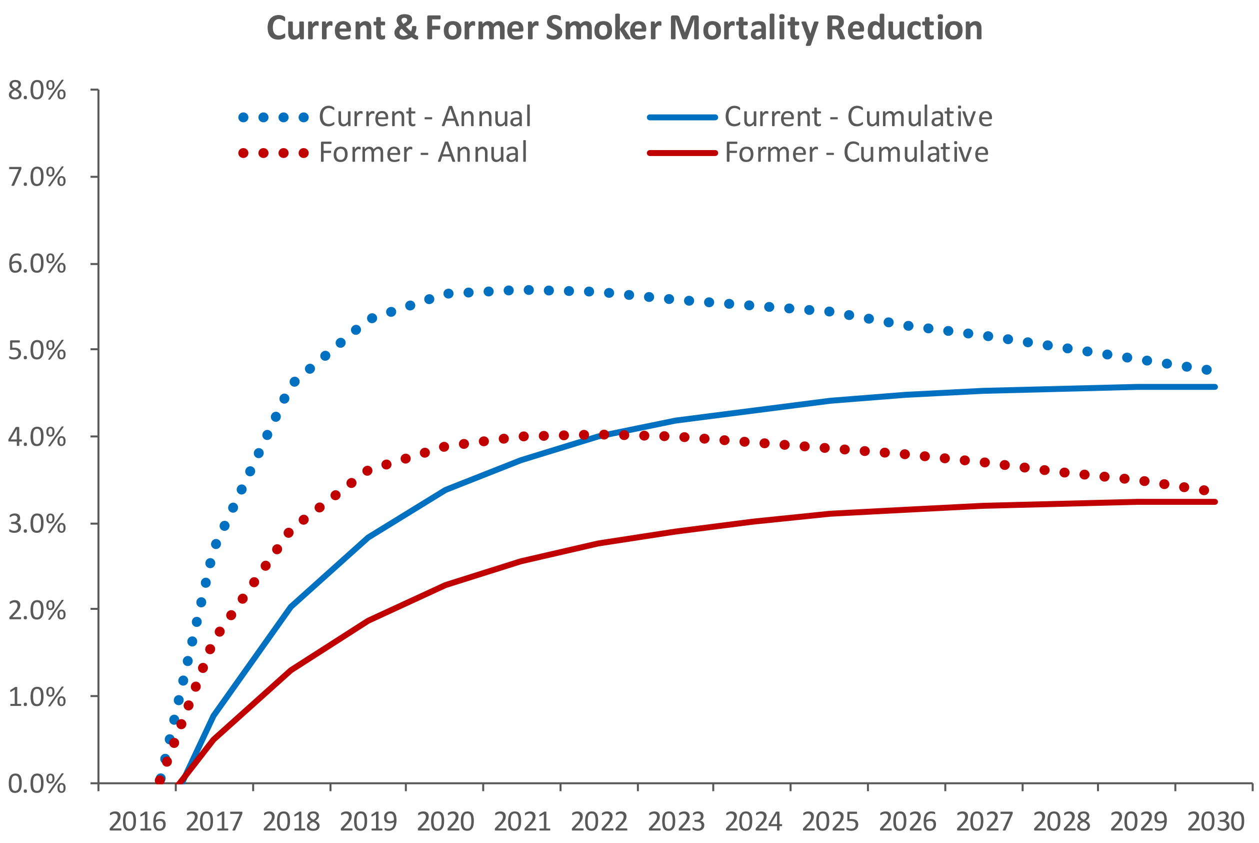

Supplementary Figure 2. Annual and cumulative mortality reduction both experience a drastic increase in the first years of the lung cancer screening program, but eventually level out as the program becomes fully implemented and decreasing smoking prevalence begins to reduce the potential benefit of screening. Current smokers derive a greater reduction in mortality than do former smokers, on both an annual and cumulative basis. After mortality reduction reaches its maximum, its extended plateau above 3% for both smoker types demonstrates the continued efficacy of lung cancer screening.

Supplementary Figure 3

Share of cumulative deaths avoided stratified by age range, 2016-2030.

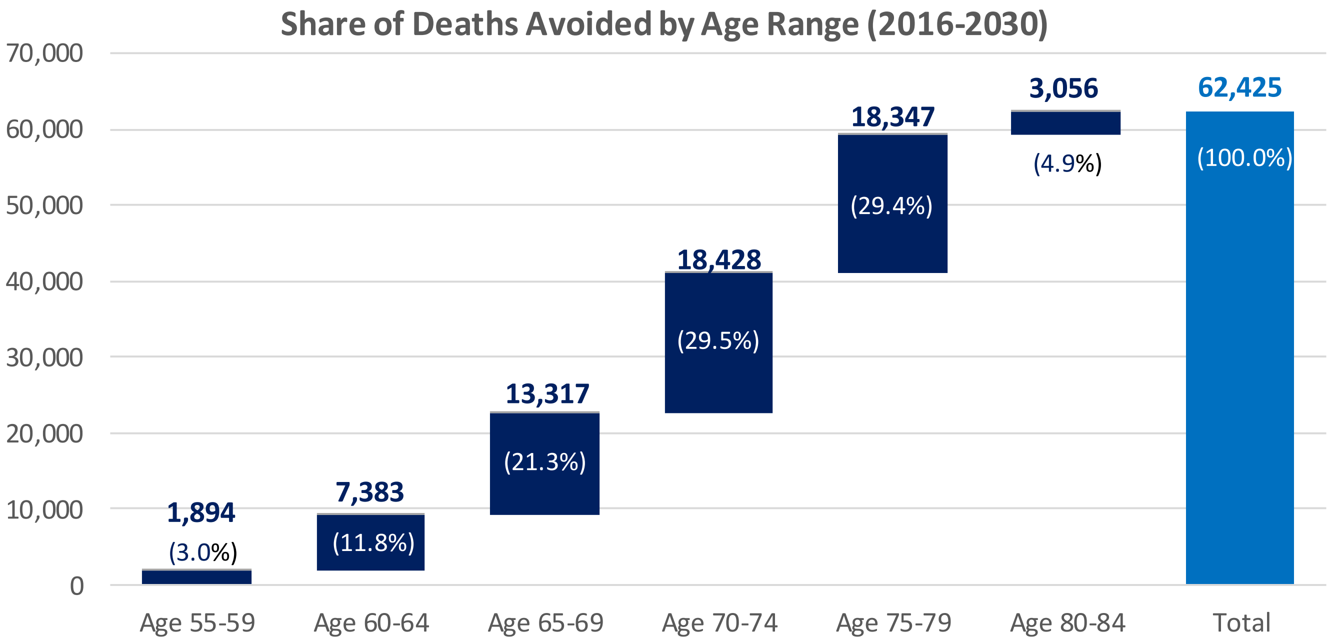

Supplementary Figure 3. Older individuals make up the largest proportion of deaths avoided, as older individuals are more likely to develop lung cancer, increasing the opportunity for early detection. The high proportion of deaths avoided that occurred between the ages of 75-79 shows that even at these older ages, lung cancer screening can provide substantial benefit. The share of deaths avoided during the ages 80-84 is much lower than the prior age range due to the fact that screening stops at 77 years old.

Supplementary Figure 4

Cumulative mortality reduction for single birth cohorts.

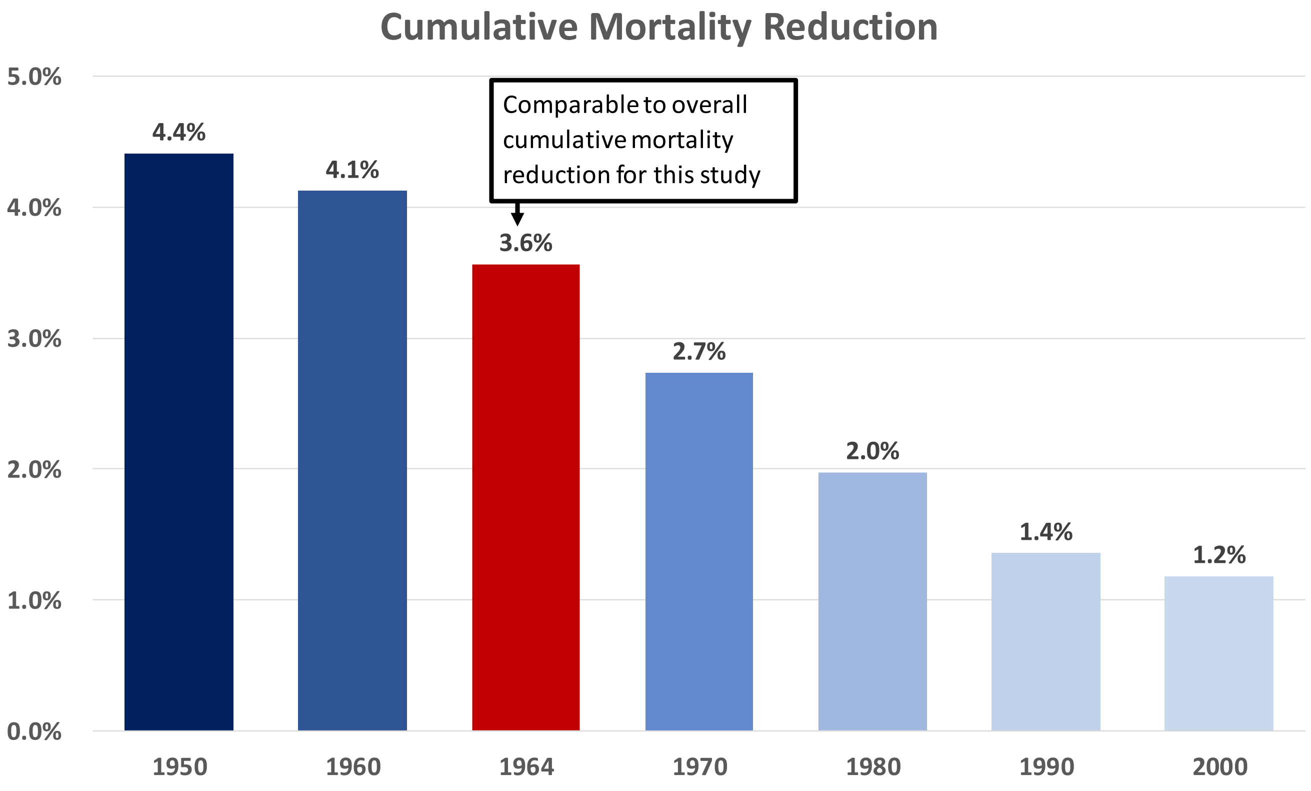

Supplementary Figure 4. Earlier birth cohorts (especially those in the 1950s and early 1960s) have higher smoking prevalence than later birth cohorts, resulting in significantly higher mortality reduction. This analysis of mortality reduction for single birth cohorts exemplifies the bias inherent to other modeling studies that only consider one birth cohort in their simulations. Smoking prevalence trends lower for each birth cohort and, therefore, later birth cohorts will limit the potential benefit of screening. By singling out one birth cohort, the results of the simulation are limited to the characteristics of that birth cohort and do not take into consideration the greater population. Studying multiple birth cohorts provides a more realistic analysis of lung cancer screening, as these trends in smoking behavior are captured. This information is more valuable to public health policy decision makers since it better represents the healthcare landscape to be addressed by lung cancer screening policy.

Supplementary Figure 5

Comparison of the Patz method of calculating overdiagnoses and the alternative method used in our study.

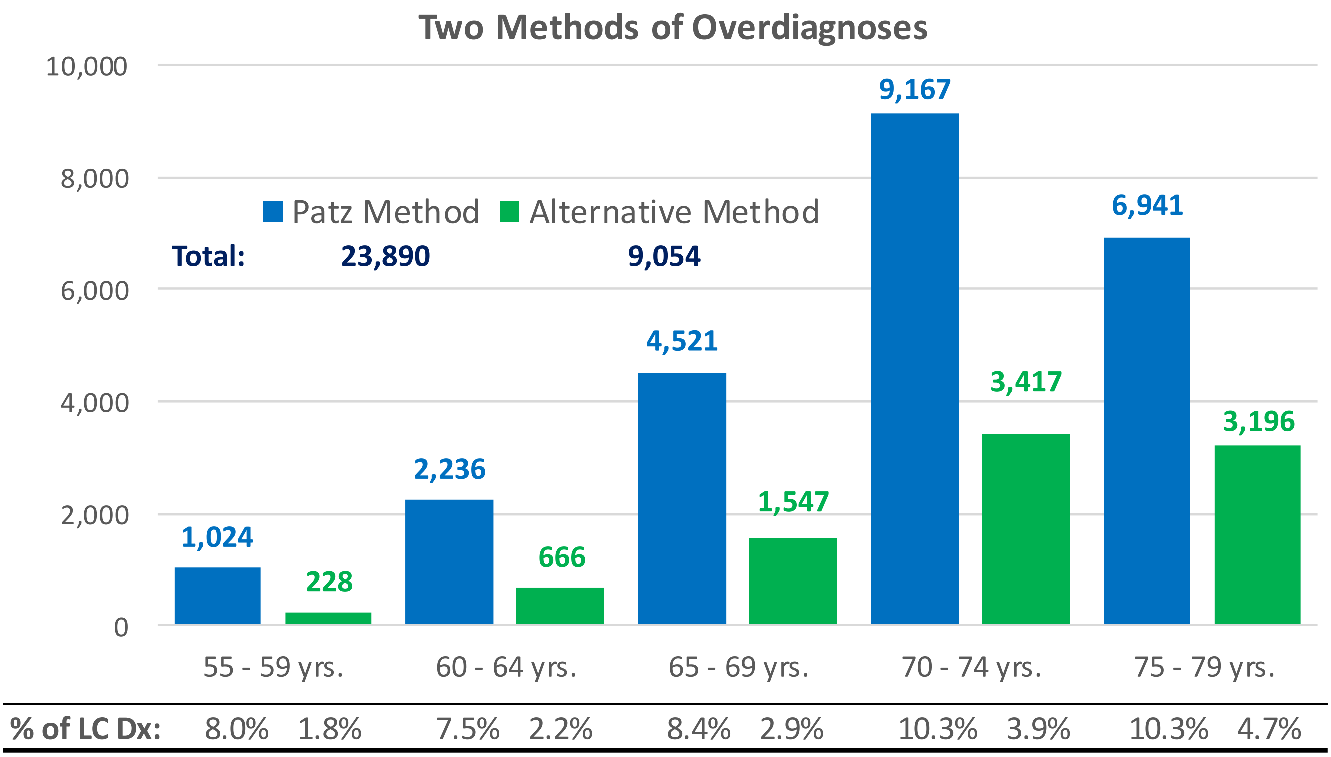

Supplementary Figure 5. Overdiagnoses using each method are given for different age ranges, with the percentage of screen-detected lung cancers made up by overdiagnoses given at the bottom. The Patz method of calculating overdiagnoses takes the number of excess lung cancer cases in the LDCT screening arm compared to the chest x-ray control arm, or in our case the no screening scenario, and divides this number by the total number of lung cancers diagnosed in the LDCT arm. The alternative method used in our study defines overdiagnoses as those in which an individual is screened and subsequently diagnosed with lung cancer in the screening scenario, but is not diagnosed with lung cancer in the no screen scenario, and eventually dies of causes other than lung cancer. The Patz method provides a much higher estimation of overdiagnoses because it includes even those additional lung cancer diagnoses in the screening arm that prevent lung cancer deaths.

Supplementary Figure 6

Study population stratified by smoker type

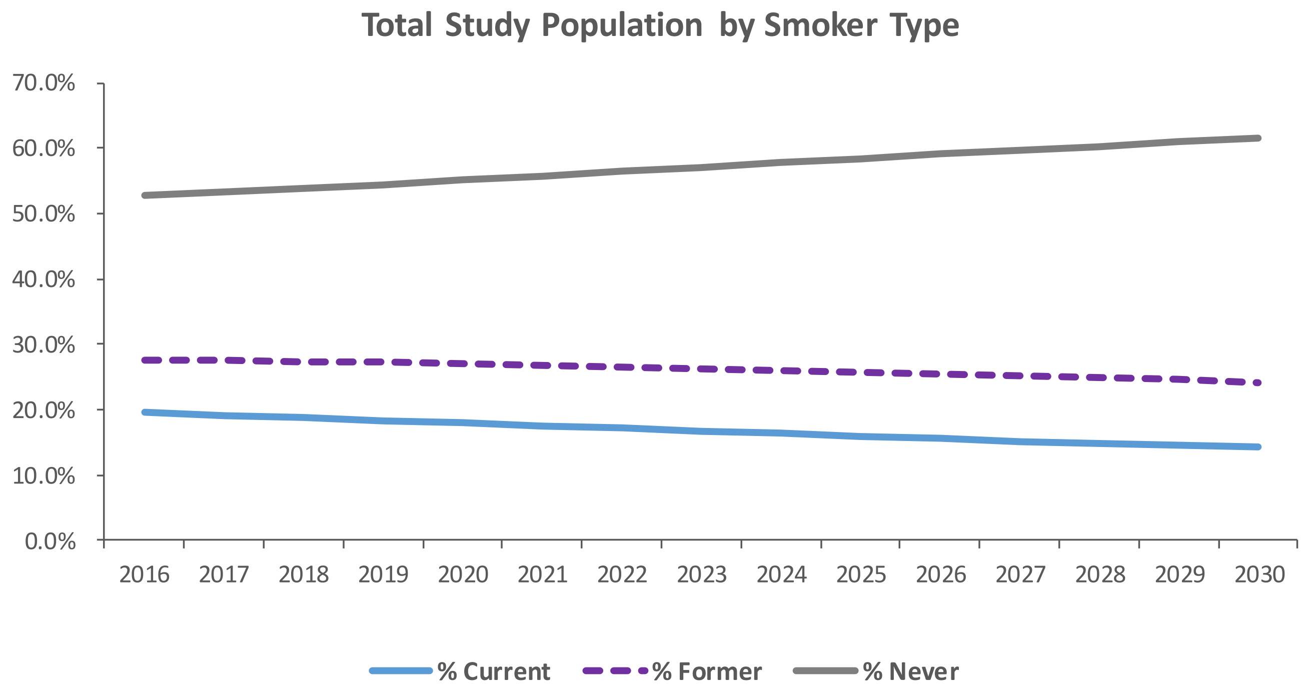

Supplementary Figure 6. Former smokers make up the largest proportion of the smoking population and a significant portion of the overall study population. The study population includes individuals age 30-84 years old in the U.S. Since former smokers make up a greater part of the population than do current smokers, overall mortality reduction more closely resembles that of former smokers (3.5% overall vs. 3.3% for former smokers and 4.6% for current smokers).
