# Supplementary material for: Population impact of lung cancer screening in the United States: Projections from a microsimulation model
Source: PLoS Med. 2018 Feb 7;15(2):e1002506. doi: 10.1371/journal.pmed.1002506 (PMC5802442; doi:10.1371/journal.pmed.1002506)

S3 Appendix

Population Impact of Lung Cancer Screening in the United States

Results using 100% Adherence Rate

Contents

Study Results Assuming 100% Screening Adherence Rate

Page 3 – **Supplementary Figure 7:** Total number of current and former smokers screened

Page 3 – **Supplementary Figure 8:** Mortality reduction compared to NLST result

Page 4 – **Supplementary Figure 9:** Mortality reduction stratified by smoker type and sex

Page 4 – **Supplementary Figure 10:** Mortality reduction for total study population on annual and cumulative basis

Page 5 – **Supplementary Figure 11:** Mortality reduction for single birth cohorts

Page 5 – **Supplementary Table 1:** Deaths avoided for current and former smokers compared to total population

Page 6 – **Supplementary Figure 12:** Percent of population screened by smoker type among 55-77 year-olds

Page 6 – **Supplementary Figure 13:** Current and former smoker mortality reduction on annual and cumulative basis

Page 7 – **Supplementary Figure 14:** Share of cumulative deaths avoided stratified by age range, 2016-2030

Page 7 – **Supplementary Figure 15:** Comparison of the Patz method of calculating overdiagnoses and the alternative method used in our study

Results: 100% Screening Adherence

Supplementary Figure 7. Total number of current and former smokers screened, 2016-2030.

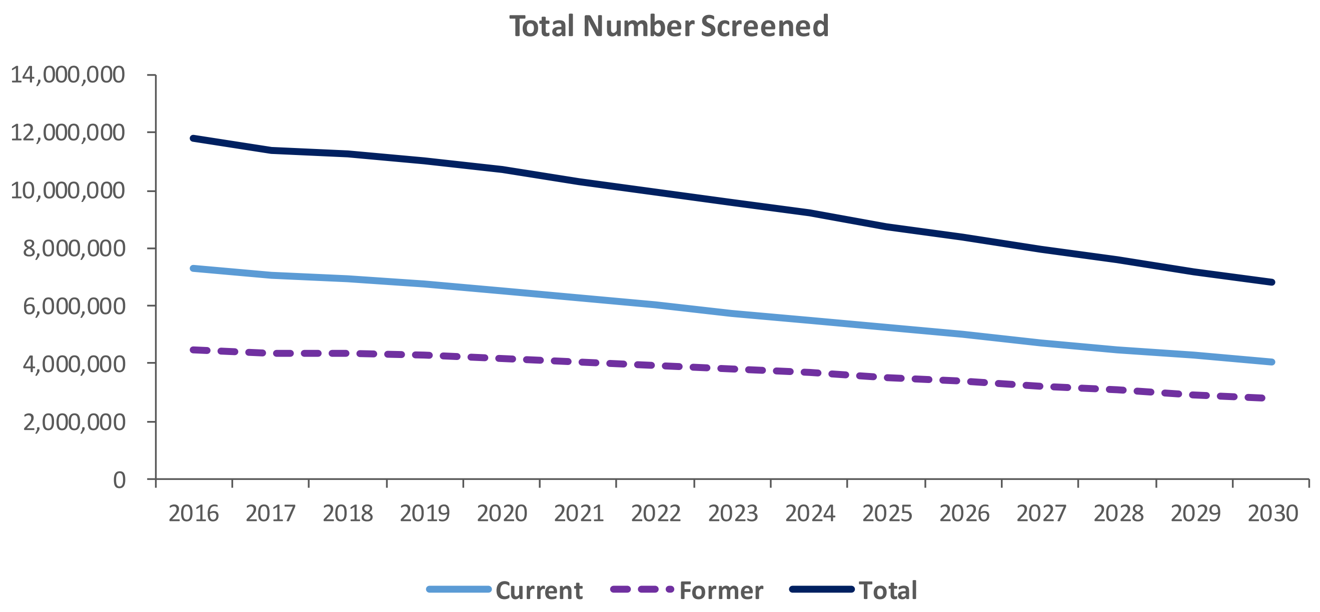

Supplementary Figure 8. Cumulative mortality reduction compared to NLST result.

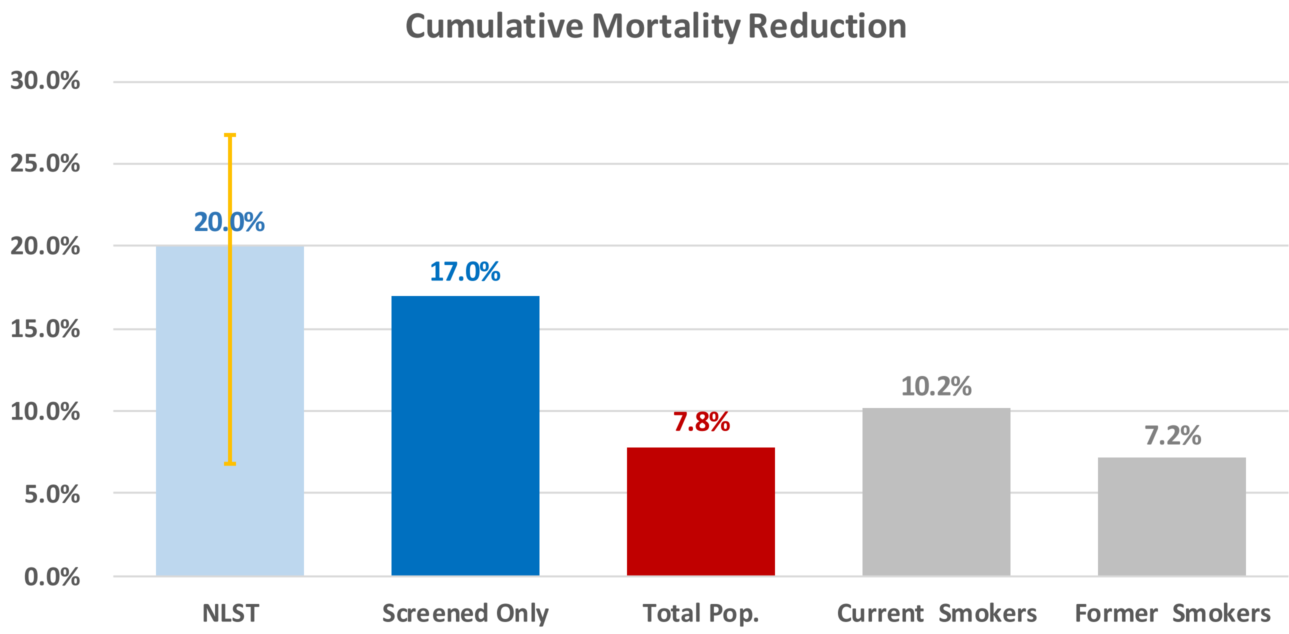

Results: 100% Screening Adherence (Cont.)

Supplementary Figure 9. Cumulative mortality reduction stratified by smoker type and sex.

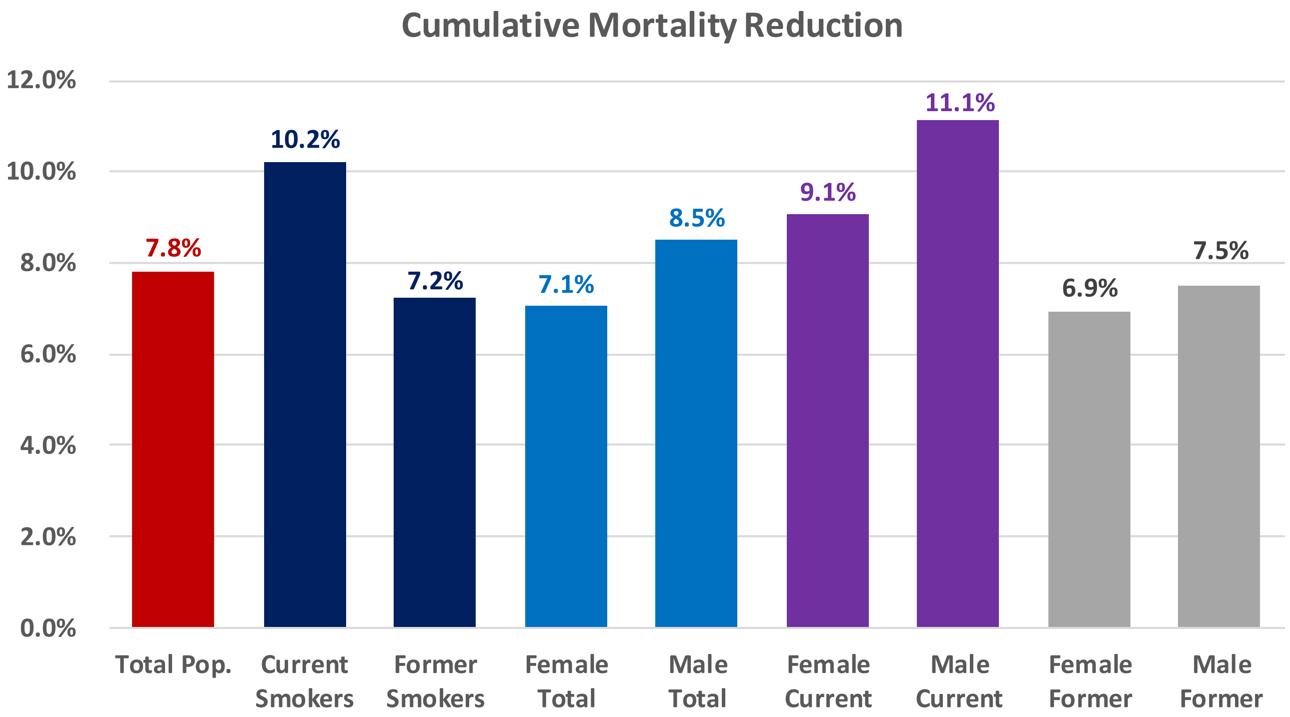

Supplementary Figure 10. Mortality reduction for total study population on annual and cumulative basis *(extended past study period to show trend).*

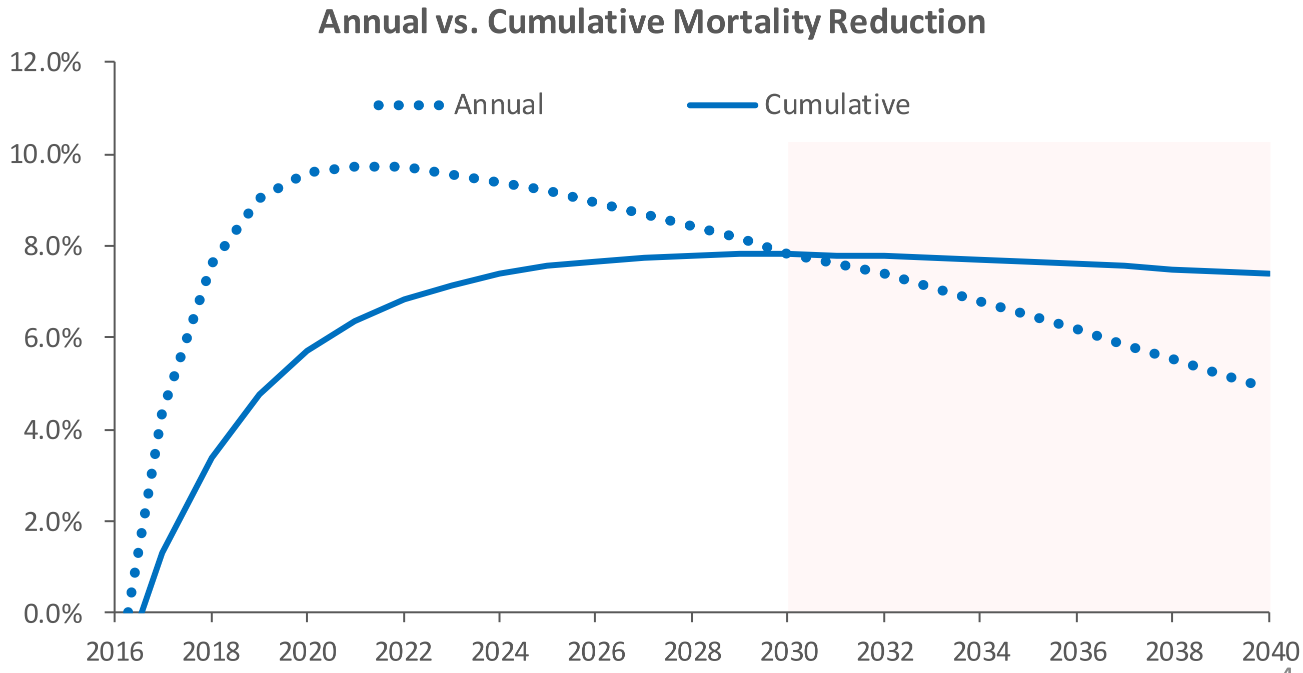

Results: 100% Screening Adherence (Cont.)

Supplementary Figure 11. Cumulative mortality reduction for single birth cohorts.

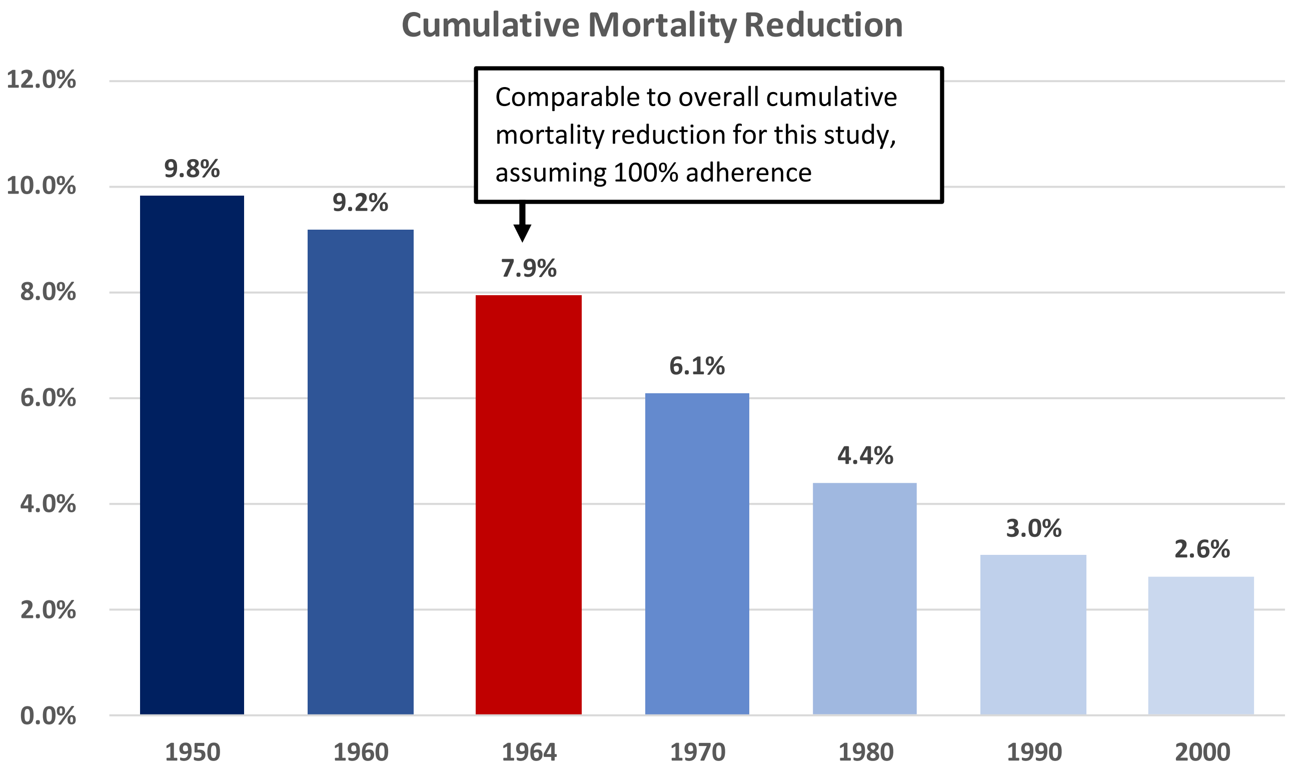

Supplementary Table 1. Deaths avoided for current and former smokers compared to total population.

Total Deaths Avoided by Smoker Type (95% CI)				
**Smoker Type**	2016-2020	2021-2025	2026-2030	**Cumulative**
**Current**	19,691 (19,488 - 19,895)	28,650 (28,427 - 28,873)	21,618 (21,420 - 21,816)	69,959 (69,380 - 70,539)
**Former**	17,189 (17,054 - 17,324)	28,444 (28,198 - 28,690)	23,130 (22,910 - 23,350)	68,763 (68,216 - 69,310)
**Total**	**36,880 (36,656 - 37,104)**	**57,094 (56,824 - 57,364)**	**44,748 (44,455 - 45,042)**	**138,722 (138,052-139,393)**

Results: 100% Screening Adherence (Cont.)

Supplementary Figure 12. Percent of population screened by smoker type among 55-77 year-olds.

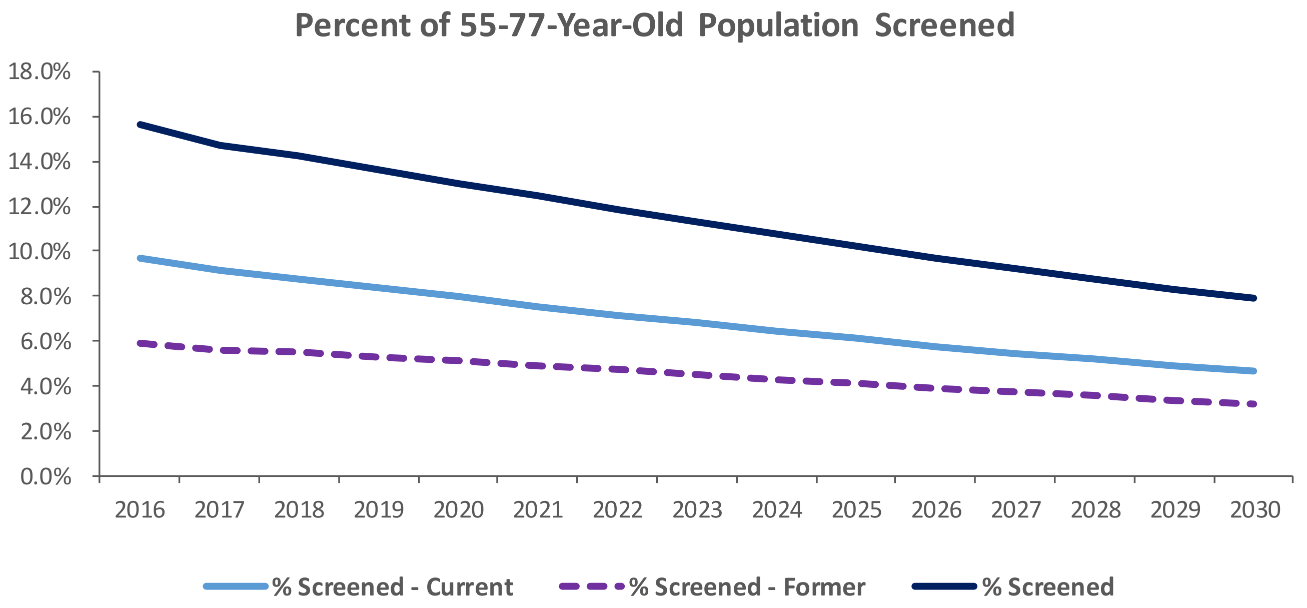

Supplementary Figure 13. Current and former smoker mortality reduction on annual and cumulative basis.

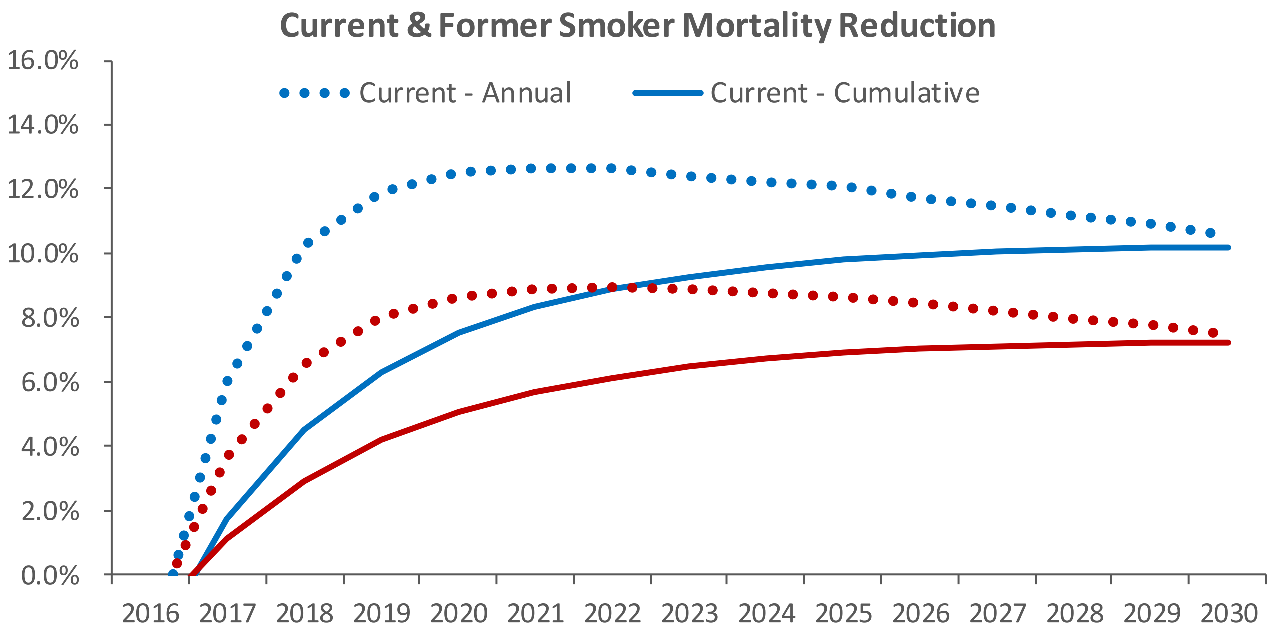

Results: 100% Screening Adherence (Cont.)

Supplementary Figure 14. Share of cumulative deaths avoided stratified by age range, 2016-2030.

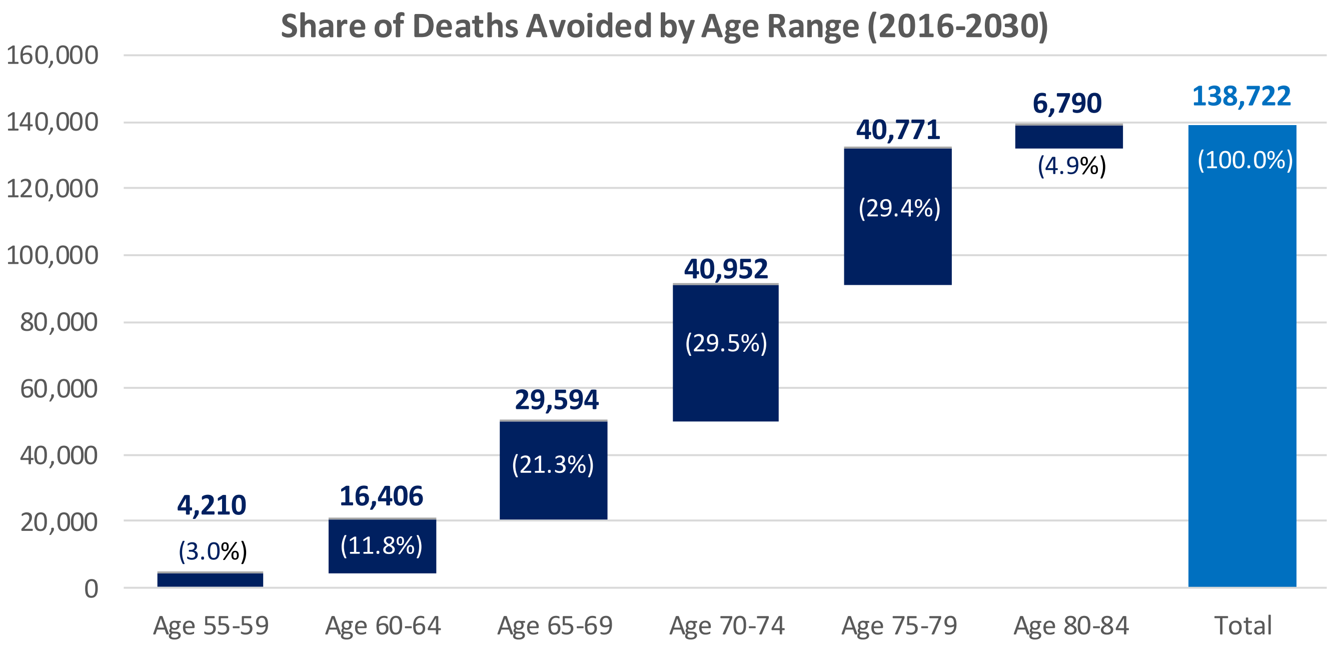

Supplementary Figure 15. Comparison of the Patz method of calculating overdiagnoses and the alternative method used in our study.

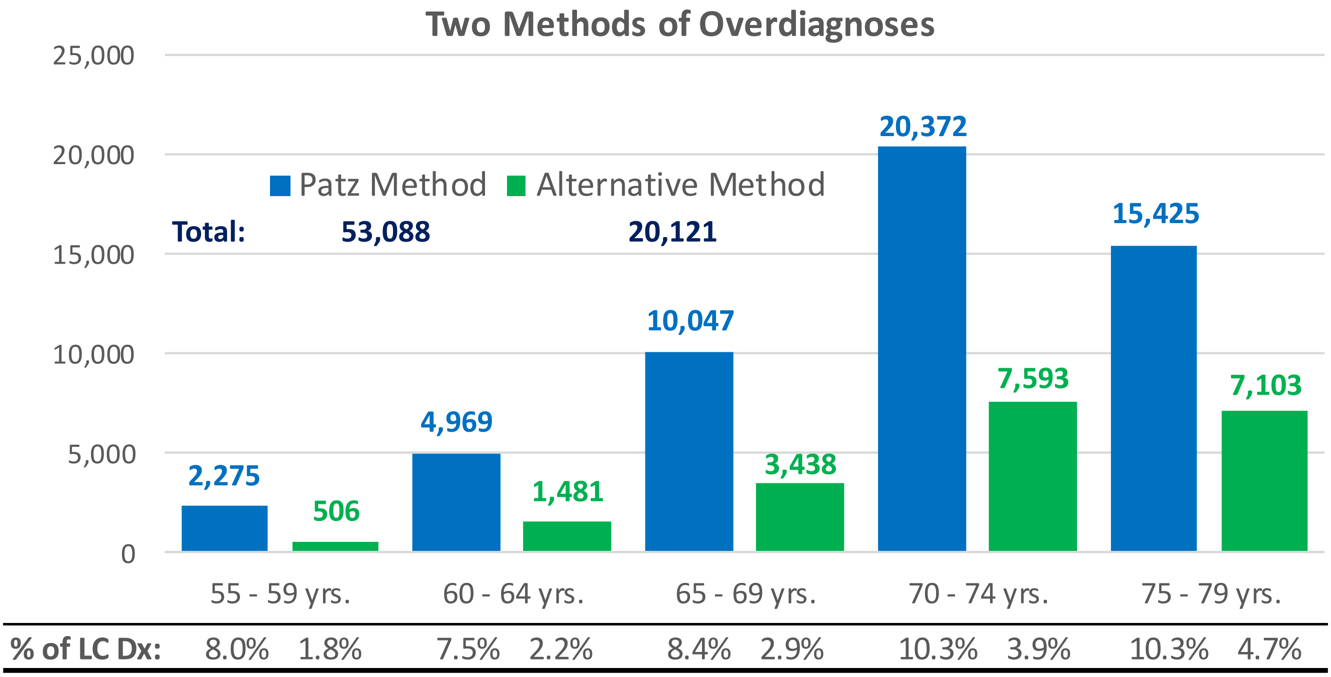

Supplement: S3 Appendix — (PDF) [file pmed.1002506.s003.pdf]
