# Supplementary material for: Population impact of lung cancer screening in the United States: Projections from a microsimulation model
Source: PLoS Med. 2018 Feb 7;15(2):e1002506. doi: 10.1371/journal.pmed.1002506 (PMC5802442; doi:10.1371/journal.pmed.1002506)
Supplement: S1 Text — (DOCX) [file pmed.1002506.s005.docx]

S1. Text: Data Availability Statement

1. Population projections from the U.S. Census Bureau used in determining future lung cancer incidence can be found at the following URL: <https://www.census.gov/programs-surveys/popproj.html>.
2. Natural history parameters for the Lung Cancer Policy Model were calibrated to National Lung Screening Trial (NLST) data. Data from the NLST can be requested from the National Cancer Institute’s Cancer Data Access System at the following URL: <https://biometry.nci.nih.gov/cdas/nlst/>.
3. Model outputs were validated using data from the Prostate, Lung, Colorectal and Ovarian (PLCO) Cancer Screening Trial. Data from the PLCO Cancer Screening Trial can be requested from the National Cancer Institute’s Cancer Data Access System at the following URL: <https://biometry.nci.nih.gov/cdas/plco/>.
4. Individual-level characteristics specific to the U.S. population were modeled using the National Cancer Institute’s Cancer Intervention and Surveillance Modeling Network (CISNET) “smoking history generator,” which is described in more detail in S1 Appendix and on the CISNET website at the following URL: <https://resources.cisnet.cancer.gov/projects/>.
